# Transient Receptor Potential Canonical (TRPC) Channels: Then and Now

**DOI:** 10.3390/cells9091983

**Published:** 2020-08-28

**Authors:** Xingjuan Chen, Gagandeep Sooch, Isaac S. Demaree, Fletcher A. White, Alexander G. Obukhov

**Affiliations:** 1 Institute of Medical Research, Northwestern Polytechnical University, Xi’an 710072, China; xjchen@nwpu.edu.cn; 2 The Department of Anatomy, Cell Biology & Physiology, Indiana University School of Medicine, Indianapolis, IN 46202, USA; gsooch@iu.edu (G.S.); idemaree@iu.edu (I.S.D.); 3 The Department of Anesthesia, Indiana University School of Medicine, Indianapolis, IN 46202, USA; fawhite@iu.edu; 4 Stark Neurosciences Research Institute, Indiana University School of Medicine, Indianapolis, IN 46202, USA

**Keywords:** TRPC, cation channels, calcium influx, TRPC modulators, TRPC knockouts

## Abstract

Twenty-five years ago, the first mammalian Transient Receptor Potential Canonical (TRPC) channel was cloned, opening the vast horizon of the TRPC field. Today, we know that there are seven TRPC channels (TRPC1–7). TRPCs exhibit the highest protein sequence similarity to the *Drosophila melanogaster* TRP channels. Similar to *Drosophila* TRPs, TRPCs are localized to the plasma membrane and are activated in a G-protein-coupled receptor-phospholipase C-dependent manner. TRPCs may also be stimulated in a store-operated manner, via receptor tyrosine kinases, or by lysophospholipids, hypoosmotic solutions, and mechanical stimuli. Activated TRPCs allow the influx of Ca^2+^ and monovalent alkali cations into the cytosol of cells, leading to cell depolarization and rising intracellular Ca^2+^ concentration. TRPCs are involved in the continually growing number of cell functions. Furthermore, mutations in the TRPC6 gene are associated with hereditary diseases, such as focal segmental glomerulosclerosis. The most important recent breakthrough in TRPC research was the solving of cryo-EM structures of TRPC3, TRPC4, TRPC5, and TRPC6. These structural data shed light on the molecular mechanisms underlying TRPCs’ functional properties and propelled the development of new modulators of the channels. This review provides a historical overview of the major advances in the TRPC field focusing on the role of gene knockouts and pharmacological tools.

## 1. Introduction and Historical Overview

The term “TRP” (Transient Receptor Potential) was coined by Minke, Wu, and Pak in 1975 [1] and referred to a spontaneous *Drosophila melanogaster* (fruit fly) mutant isolated by Cosens and Manning in 1969 [2]. This mutant of *Drosophila melanogaster* exhibited apparently normal vision under low ambient illumination but behaved as blind following exposure to a bright light for a time longer than 5–10 s. Cosens and Manning attributed this defect to the abnormal electroretinogram of the mutant’s compound eye. The wild type compound eye electroretinogram was characterized by a sustained depolarization or receptor potential during a prolonged bright light illumination, whereas the mutant fly compound eye electroretinogram revealed only a transient receptor potential under the same prolonged bright light stimulation [2]. Using the break point analysis, the *trp* mutation was mapped to a locus on the third chromosome of the *Drosophila* fly genome by Wong et al. in 1985 [3]. Subsequently, the Drosophila *trp* gene was cloned in 1989 by two independent groups of Montell and Rubin [4] and Wong et al. [5].

Based on the TRP protein sequence, Montell and Rubin (1989) [4] predicted that the *trp* gene may encode a 1275 amino acid protein with eight transmembrane segments, typical for some cation channels, but the hypothesis that the TRP protein may be a transporter could not be ruled out. Only in the seminal 1992 work by Roger Hardie in collaboration with Baruch Minke [6] was the first experimental evidence provided indicating that the TRP protein forms a light-sensitive channel required for inositide-mediated Ca^2+^ entry in Drosophila photoreceptor cells. In the same year, Phillips et al. [7] identified a *Drosophila* homolog of TRP, the TRP-like (TRPL) gene encoding a calmodulin-binding protein. It was later demonstrated by Niemeyer et al. (1996) and Reuss et al. (1997) that the presence of TRPL allowed the *Drosophila trp* mutant fly to see in dim light [8,9]. Further evidence supporting the fact that TRPL forms a channel was provided in 1996 when the Günter Schultz laboratory published the recordings of single-channel activity of the TRPL channel induced by the purified Gq protein stimulating phospholipase C (PLC) in isolated inside-out patches [10]. This was the first recording of single-channel activity of a TRP channel. Later, the same group used single-channel recordings to demonstrate that the TRPL channel is inhibited by intracellular Ca^2+^ [11]. The fact that the TRP protein forms a channel was further supported by evidence obtained in an in vivo study of the Roger Hardie laboratory in collaboration with Obukhov and Montell [12]. This work demonstrated that the Drosophila TRP channel’s selectivity filter residue Asp621 in the putative pore loop is the major molecular determinant of *Drosophila* TRP channel selectivity to Ca^2+^ [12]. There are many reviews recapping the history of *trp* discovery, but the most accurate account of those events was provided by Roger Hardie [13]. 

Importantly, the TRP channel was identified as inositide-dependent because the phototransduction process in *Drosophila* flies absolutely required the activation of the G-protein-PLC evoked phosphatidylinositol signaling [6,14,15]. While the *trp’s* history was unfolding, Jim Putney came forward with the capacitative Ca^2+^ influx model in 1986 [16]. He explained the biphasic nature of hormone-activated Ca^2+^-mobilization in cells by suggesting that inositol-1,4,5-triphosphate (IP_3_) controls both the initial rapid Ca^2+^ release from the endoplasmic reticulum, intracellular Ca^2+^ stores, and the following Ca^2+^ entry occurring due to IP_3_-dependent depletion of those intracellular Ca^2+^ stores [16]. The Putney model provided a critical blueprint, unlocking the major testing ground and fueling the progress in the receptor-operated Ca^2+^ signaling field and eventually culminating in the discovery of the Orai family of store-operated channels and STIM proteins serving as the endoplasmic reticulum’s Ca^2+^ sensors (for review see [17]). In 1991, Minke and Selinger applied the Putney capacitative model to the TRP field, proposing that store-depletion might be involved in regulating TRP protein activity [18]. Since then, this hypothesis has been attracting much interest in the Drosophila TRP field, with some studies supporting it [19,20], and others contesting it [21]. 

Cloning of TRP and TRPL genes allowed the researchers to employ various expression models while investigating the proteins’ functions. This further stimulated the progress in the field. The pioneering works were performed in the laboratory of Bill Schilling in 1994. His research team demonstrated, using the SF9 insect expression model, that the TRPL channel was constitutively active [22], whereas the TRP channel could be activated by store-depletion [23]. In 1995, the laboratories of Günter Schultz and Schilling independently reported that TRPL can be activated downstream of the Gq-protein coupled receptor signaling [24,25]. This was a major step towards understanding the mechanisms of the TRPL channel activation. However, the store-operated mechanism was still dominating the field. The Minke group reported that the coexpression of both Drosophila TRP and TRP-like proteins was required for reconstituting capacitative Ca^2+^ influx in Xenopus oocytes [26]. Consistently, the Montell group provided evidence that the endogenous dTRP can heteromerize with dTRPL to form store-operated channels [27], concluding that the light-activated current in Drosophila photoreceptor cells results from a combination of TRP homo- and TRP–TRPL heteromultimers. 

## 2. Discovery of TRPC Channels

**Figure 1:**
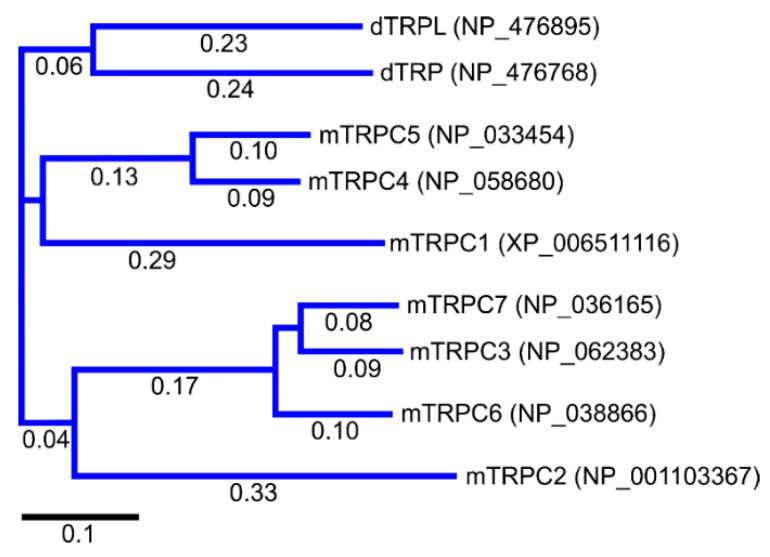
The phylogenetic tree of *Drosophila melanogaster* TRP channels and mouse TRPC channels. The multiple sequence alignment was performed using the MUSCLE algorithm. The length of branches is shown under the lines, indicating the number of substitutions per site. The scale bar is also included under the plot for convenience. Based on the phylogenic tree, mouse TRPCs can be subdivided into four groups: TRPC1, TRPC2, TRPC4/5 and TRPC3/6/7. MegAlign Pro 17 of Lasergene 17 software was used to align and construct the tree. The TRP protein accession numbers are shown on the right from the name of each protein.

The importance of store-operated/capacitative Ca^2+^ influx in human immune cells fueled the race for cloning the mammalian homologs of *Drosophila* TRP. The first mammalian TRP channel homolog, TRPC1, was cloned in 1995, exactly 25 years ago, by two independent groups of Montell [28] and Birnbaumer [29]. The other six members of the TRPC subfamily were cloned soon after (TRPC3 [30]; TRPC2 [31]; TRPC4 [32]; TRPC5 [33,34]; TRPC6 [35]; TRPC7 [36,37]). Because of TRPCs’ high similarity to *Drosophila* TRPs (Figure 1), the TRPC properties and function were often viewed through the lenses with a “*Drosophila TRP tint*”. Indeed, many of those newly cloned channels were first characterized as store-operated channels (TRPC1 [30,38]; TRPC3 [30]; TRPC4 and TRPC5 [32,33]; TRPC7 [37]). Only the TRPC6 channel was initially reported to be activated in a receptor-operated manner [35]. However, it was later demonstrated that both TRPC3 and TRPC6 can be activated by the agonists of G-protein coupled receptors in a PLC-dependent, but store-depletion independent manner [39,40]. Additionally, TRPC4 and TRPC5 channels were also later reported to function independently of store-depletion [34,41], but being activated via G-protein coupled receptors or receptor tyrosine kinases downstream of PLC. Thus, as for the *Drosophila* TRP channels, no consensus was reached regarding the mechanism of TRPC channels’ activation. However, everyone agreed that activation of all TRPC channels results in a rise of intracellular Ca^2+^ concentration and/or the membrane potential depolarization. 

The TRPC subfamily contains seven members, TRPC1–7 (Figure 1). Based on sequence homology, TRPC channels were further subdivided into four subgroups: TRPC1, TRPC2, TRPC4/5, and TRPC3/6/7 subfamilies. TRPC2 channel is not expressed in humans [42], therefore this channel is not discussed in this review. While the race to clone all of the TRPC channels was going on, many research groups rushed to establish the mechanisms leading to TRPC channel activation. Using the treasure trove of data accumulated for homologous *Drosophila* TRP channels, TRPCs were expected to be activated either in a store-operated manner or following stimulation of the G-protein coupled receptor-PLC signaling. Because PLC hydrolysis of phosphatidylinositol 4,5-biphosphate (PIP_2_) results in the production of IP_3_ and diacylglycerol (DAG), the role of these two second messengers in activation of newly cloned TRPC channels was scrutinized first. 

Initial reports from the Günter Schultz group provided evidence that TRPC1 functions as a store-operated channel (1996 [38]), whereas the TRPC3 channel is a receptor-operated channel that can be activated in a store-depletion independent manner (1997 [39]). The proposed TRPC3 activation mechanism was soon challenged by the Muallem group who reported in 1998 that human TRPC3 can be activated by store-depletion or direct interaction with the IP_3_-bound IP_3_-receptor [43]. Three years later, the Mike Zhu group demonstrated that TRPC3 and all other TRPCs contain a calmodulin/IP_3_-receptor-binding (“CIRB”) domain at the channels’ C-terminus, and Ca^2+^-calmodulin bound to this CIRB domain inhibits TRPC3 channel activity, whereas the displacement of the inhibitory calmodulin from the CIRB site by the IP_3_R may activate the TRPC3 channel [44,45].

However, at the same time, TRPC3, TRPC6, and TRPC7 channels were also demonstrated to be directly activated by DAG or 1-oleoyl-2-acetyl-sn-glycerol (OAG) in a PKC-independent manner [36,40,46]. Conversely, TRPC4 and TRPC5 were initially identified as insensitive to either DAG or Ca^2+^ store-depletion [41]. Importantly, it was shown that TRPC3 and TRPC5 channels can be activated in a PLC-dependent but IP_3_-receptor- or Ca^2+^ store-depletion independent manner when expressed in DT40 B cells lacking all of the intracellular IP_3_-receptors [47], indicating that the IP_3_R is not necessary for these specific TRPC channel activation. A complex relationship between PIP_2_ degradation and TRPC3, TRPC6, and TRPC7 channel activation by DAG was reported [48,49]. Trebak et al. [49] established that, although PIP_2_ degradation is important for TRPC3, TRPC6, and TRPC7 channel activation by DAG, it was also necessary for the closing of TRPC3, TRPC6 and TRPC7 channels. Remarkably, the DAG binding site on TRPC3 has been recently mapped using a structure-guided mutagenesis strategy which revealed the existence of a critical glycine residue behind the selectivity filter (G652) regulating DAG-sensitivity of the channel and accessible through a subunit-joining fenestration [50]. 

Based on the sensitivity to DAG, the TRPC channels were initially subdivided into two subgroups, the DAG-insensitive TRPCs, such as TRPC1, TRPC4 and TRPC5, and the DAG-sensitive TRPCs, including TRPC3, TRPC6 and TRPC7. It was later reported that TRPC4 and TRPC5 channels can interact and be functionally modulated with/by Na^+^/H^+^ exchanger regulatory factor 1/2 (NHERF1/2) [51,52], and NHERF1/2 interaction with TRPC4 and TRPC5 requires the channels C terminal VTTRL motif [51]. The Gudermann group established in 2017 that interaction of NHERF1/2 with the C terminus of TRPC4 and TRPC5 is responsible for the lack of DAG sensitivity of TRPC4 and TRPC5 channels [53], while PIP_2_ depletion and PKC inhibition may promote dissociation of NHERF1/2 from the TRPC4′S and TRPC5′s C-termini conferring DAG sensitivity to the channels [53]. A C-terminal residue T^972^ was identified to be phosphorylated by PKC in TRPC5, making the channel insensitive for DAG stimulation if phosphorylated. Thus, the authors of [53] argued that all TRPC channels should be considered as PLC-dependent and DAG sensitive channels. 

Whether TRPC1/TRPC4/TRPC5 are activated via the PLC-dependent mechanism without store-deletion has been long debated. Some groups provided supporting evidence for the store-independent activation [41,47,54,55,56], whereas others argued that the store-operated mechanism is the major route for the channels’ activation [38,57,58]. Neither DAG nor IP_3_, nor the combination of the two signaling molecules, could stimulate TRPC1/TRPC4/TRPC5 currents [41,47]. As in *Drosophila* photoreceptors, IP_3_ is responsible for Ca^2+^ release from its intracellular store in mammalian cells that may result in store depletion. Notably, the TRPC1 channel was shown to be activated by store-depletion [38,58] and evidence was later provided that Orai/STIM1 proteins’ interaction with TRPC3 and TRPC6 [59] or TRPC1 [60] may confer a greater sensitivity to store depletion to the channels. 

TRPCs can also be activated in a PLC-independent manner. Lysophospholipids were reported to activate TRPC6 [61] and TRPC5 [62] channels. Sphingosine-1-phosphate can activate TRPC5 and TRPC5–TRPC1 heteromeric channels while regulating smooth muscle cell motility [63]. TRPC5 channel activity can be stimulated by perfusion of extracellular hypoosmotic solution or pressure-induced membrane stretch [64], whereas TRPC6 channel activity may be induced by H_2_O_2_ via a cysteine oxidation-dependent pathway that not only activates the channel, but also sensitizes it to DAG-activation and promotes its trafficking to the plasma membrane [65]. Spassova et al. [66] provided evidence that TRPC6 may function as a mechanosensitive, stretch-activated channel and proposed a model of TRPC6 activation involving changes in plasma membrane geometry. According to this model, TRPC6 opens either by stretch-induced plasma membrane thinning or due to a PLC-dependent PIP_2_ breakdown to DAG resulting in the drastic change in local membrane curvature. PIP2 enrichment in the plasma membrane is indeed associated with positive membrane curvature, whereas increased plasma membrane DAG concentration promotes negative membrane curvature [67]; however, a study by Hirma et al. [68] showed that anionic phospholipid membrane curvature can be significant only after complete depletion of cholesterol in the plasma membrane that is unlikely to occur under physiological conditions. It was also reported that TRPC1 can be activated by membrane stretch [69]. However, this finding was later contested in a TRPC1 KO mouse model, in which pressure-induced and store-operated cation influx in vascular smooth muscle cells was unaltered [70]. Although it was confirmed that neither TRPC1 nor TRPC6 contributes to mechanosensitive currents in the COS cell overexpression model [71], more recent data in native primary cultures provided evidence that TRPC1 and/or TRPC6 may be important for mechanosensitivity of neurons [72], specifically for the detection of innocuous mechanical force [73] or primary afferent nociceptor sensitization while cooperating with osmosensitive channels such as TRPV4 [74]. 

The TRPC3/6/7 and TRPC1/4/5 subgroups exhibit several dissimilar properties. TRPC4 and TRPC5 currents are potentiated by acidic pH. Conversely, TRPC6 is inhibited by protons [75]. Additionally, TRPC4 and TRPC5 are potentiated by La^3+^ and Gd^3+^ [41,54,56,76], whereas TRPC6 is inhibited by lanthanides [54]. 

It was already noted above that *Drosophila* TRP and TRPL are capable of forming heteromers [27]. Consistently, TRPCs can also form heteromeric channels. The Clapham group was the first to demonstrate that TRPC1 and TRPC5 channels may form heteromeric channels exhibiting altered biophysical properties [77]. Specifically, Strubing et al. reported that the heteromeric channels had significantly smaller inward currents compared to TRPC5 homomeric channels due to markedly decreased unitary single-channel conductance at physiological membrane potentials [77]. This result was confirmed later by several independent groups [78,79]. The Clapham group also provided evidence that heteromeric proteins may be formed between TRPC subunits from different subgroups, including TRPC1–(TRPC4/TRPC5)–(TRPC3/TRPC6) heteromeric channels, at least in the mammalian brain [80]. 

Thus, the TRPC proteins form non-selective cationic channels, that are localized to the plasma membrane and enable entry of Ca^2+^ and Na^+^ into various types of cells. When activated, the channels cause membrane depolarization due to cation influx. It appears that one of the major functions of TRPCs is to convert the circulating hormonal signals into intracellular Ca^2+^ changes and cell depolarization. The TRPC proteins are widely expressed throughout the mammalian tissues, with some of TRPCs exhibiting a restricted expression pattern while others being ubiquitously expressed [81,82,83,84,85,86,87]. For example, TRPC1 is broadly expressed [28,88]. Conversely, TRPC6 is found in the tissues containing smooth muscle cells such as blood vessels, stomach, colon, lungs, and myometrium [81,89,90]. TRPC5 is highly expressed in the brain [34] and adrenal medulla [91,92]. 

Several TRPC mutations are reported to be linked to human disease (for review, see [93]). Familial focal segmental glomerulosclerosis is the most well-investigated human disease associated with multiple mutations in the TRPC6 gene [94,95]. A single nucleotide polymorphism in the TRPC6 gene may also cause idiopathic pulmonary hypertension [96]. Because of their wide expression and ability to regulate the intracellular Ca^2+^, it is not surprising that the TRPC channels have substantial importance in mammalian physiology. 

## 3. Structure of TRPC Channels

### 3.1. General Structural Organization of TRPCs

The revolutionized cryo-electron microscopy has recently allowed the determination of high-resolution structures of TRPC channels. Thus far, the structures have been solved for the hTRPC3 protein [97,98,99], the hTRPC6 protein [99,100], the mTRPC4 protein [101,102], and the mTRPC5 protein [103]. The reported TRPC structures have variable resolutions ranging from 2.8–5.8 Å, with the mouse TRPC5 structure [103] being solved at the highest resolution of 2.8 Å. The full length human TRPC3 structure has been determined at the resolutions of up to 5.8 Å [97,98,99]. The structure of the cytoplasmic domain of TRPC3 was solved at a resolution of 4.0 Å [98]. The full length and cytoplasmic domain of TRPC6 structures have been reported at a resolution of 3.8 Å [99,100]. The structures of zebrafish and mouse TRPC4 were determined by two independent groups and reported at overall resolutions of 3.6 and 3.3 Å, respectively [101,102]. These TRPC structures shed light on the channels’ inter- and intra-subunit interactions and offered insight into the general architecture and domain organization of the TRPC channels, indicating that both the specific transmembrane and cytosolic domain residues contribute to stabilizing the tetrameric organization of the channels. However, thus far, most of the reported TRPC structures are determined in a closed channel state. Only the TRPC5 structure was solved in a partially open state [103], consistent with the earlier observation that TRPC5 can exhibit spontaneous activity without receptor activation [41]. 

**Figure 2:**
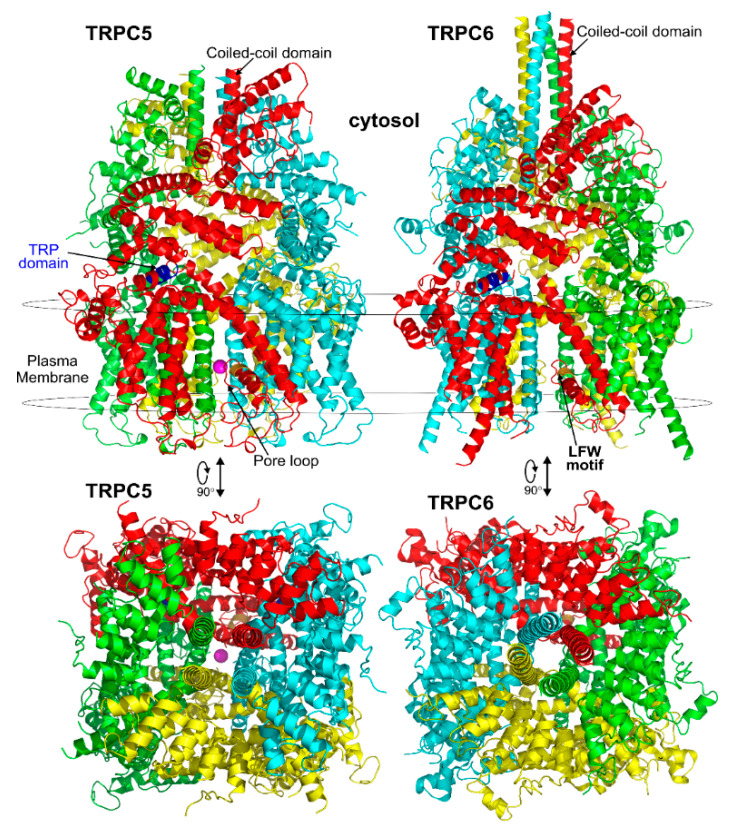
Cryo-EM structures of TRPC5 and TRPC6 channels. Each subunit in these TRPC structures was color-coded as red, green, yellow, and cyan for better identification. The TRP domain conserved in all TRPC channels is colored in blue in the red subunits of the shown structures. The LFW motif, which is located in the pore helix and colored in bright orange in the figure, is critical for the function of all TRPC channels because this protein segment is important for positioning the pore loop of the channel. Substituting AAA for LFW residues in a TRPC subunit renders it as a dominant negative. Dominant negative subunits are able to quench the activity of the functional subunits in heteromeric TRPC channels, which is a useful strategy to study TRPC channel roles in various organ systems. The mouse TRPC5 and human TRPC6 atomic coordinates were from PDB ID#: 5AEI and PDB ID#: 5YX9, respectively. A Na^+^ cation in the selectivity filter of TRPC5 channels is shown as a magenta sphere.

All of the solved cryo-EM structures confirm that each TRPC channel consists of four subunits (Figure 2) with a four-fold symmetry. Each transmembrane domain contains six ⍺-helices. The four pore loops (Figure 2) make the selectivity filter of the channel. The cytosolic C- and N-termini form a square base dome-like structure topped with a cupola lacking its roof and facing the cytosol (Figure 2). The N-terminus of TRPC channels consists of four ankyrin repeats followed by several α-helical segments (seven in TRPC4/5 and nine in TRPC3/6), whereas the C-terminus contains the short TRP domain (“EWKFAR”; Figure 2, colored in blue) followed by a relatively long connecting helix attached to a coiled-coil domain. Four coiled-coil domains form a square tube directly connecting the channel’s cytosolic dome inner vestibule and the cytosol (Figure 2). These four coiled-coil domains along with the four connecting helixes are critical for stabilizing the TRPC channels’ tetrameric structure.

### 3.2. The Pore Region

All of the reported TRPCs structures were solved in the closed conformation, except for TRPC5, which was in a partially open conformation. In the middle of the conduction pathway, there is a wide central vestibule. The narrowest point of TRPC’s cation conduction pathway forms the lower gate of the channel (e.g., L654 and I658 in TRPC3 [97]; “INQ”, Ile^621^, Asn^625^, and Gln^629^ in mTRPC5; and Ile^617^, Asn^621^, and Gln^625^ in TRPC4 [103]). All of the TRPC channels share the conserved LFW motif located within the pore helix which interact with s5-s6 helices. A π–π interaction between Phe576 and Trp577 in TRPC5 stabilizes the key pore loop region in the channel structures. The LFW-AAA triple TRPC mutant serves as a dominant negative subunit that is very effective in decreasing functional activity of heteromeric TRPC channels as verified in several studies (e.g., [80]). 

**Figure 3:**
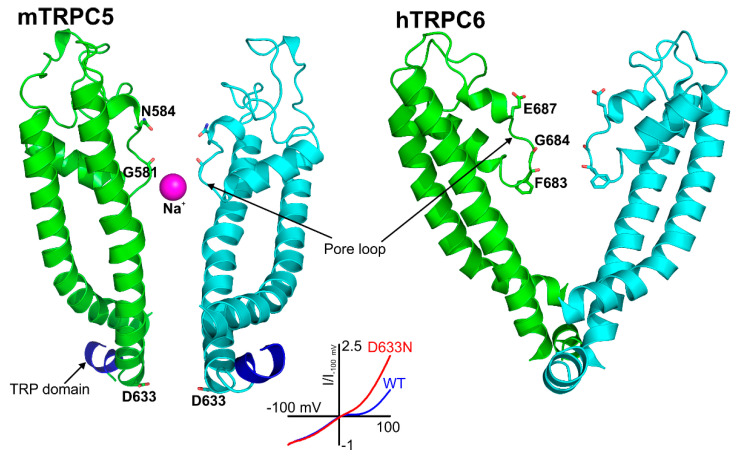
Structural architecture of the pore region of TRPC5 and TRPC6 channels. Only two subunits are shown for clarity (mouse TRPC5-pdb: 5aei and human TRPC6-pdb: 5yx9). The residues involved in controlling TRPC5 and TRPC6 cation selectivity are indicated within the pore loop of the channels. The role of the E687 residue in controlling TRPC6 Ca^2+^ permeability was identified by the Klaus Groschner group in 2011 [104,105], whereas the importance of the N584 residue for determining the TRPC5 channel’s Ca^2+^ selectivity was identified in a screen by Chen et al. in 2017 [56]. TRPC5 is inhibited by intracellular Mg^2+^ in a voltage-dependent manner, with a S6 transmembrane helix residue, D633, being responsible for that signature property of TRPC5 [79]. The inset shows the current–voltage relationships of wild type and the D633N mutant of TRPC5. The D633N mutant exhibits a reduced Mg^2+^ sensitivity, whereas the D636N mutant has a current–voltage relationship similar to that of the wild type TRPC5 [79]. The solved structure of TRPC5 confirmed that the D633 residue is located within the cation conduction pathway, whereas neighboring D636 residue faces away [103]. The mouse TRPC5 and human TRPC6 atomic coordinates were from PDB ID#: 5AEI and PDB ID#: 5YX9, respectively.

The TRPC5 channel is inhibited by intracellular Mg^2+^ at membrane potentials between 0 and +40 mV, a signature property of the TRPC5 channel (Figure 3, *inset*). The responsible residue, D633, was determined using site-directed mutagenesis [79]. Asparagine substitution for aspartate in the 633 position of TRPC5 markedly decreased the channel block by intracellular Mg^2+^, eliminating the S-shaped segment on the current–voltage relationship of the mutated TRPC5 channel. Based on the D633N phenotype, Obukhov et al. (2005) proposed that the cation conduction pathway of TRPC5 extends farther than it was predicted by the Kyte and Doolittle hydrophobicity analysis [79]. The solved structure of TRPC5 confirmed that the D633 residue is located within the cation conduction pathway, whereas a neighboring D636 residue, mutation of which to asparagine is reported not to affect the S-shape of TRPC5 current–voltage relationship [79], faces away [103]. 

The selectivity filters are well-resolved in all TRPC structures. The position of the selectivity filter is historically determined by identifying the narrowest part of the channel conduction pathway. The TRPC’s selectivity filters usually contain a glycine followed by phenylalanine in TRPC3 and TRPC6 channels [97,99] or only glycine in TRPC4 and TRPC5 channels [101,102,103]. However, since the available channel structures are solved in the closed conformation, it is unclear whether the identified narrowest pore segment is indeed the selectivity filter. During channel openings, there may be significant alteration in the position of pore loop residue side chains and the protein loop backbone. Remarkably, the residues regulating TRPC’s Ca^2+^ selectivity were determined by mutagenesis. The importance of the E630 residue, preceding the putative TRPC3 selectivity filter, in controlling the Ca^2+^ permeability of human TRPC3 channel was first demonstrated by the Klaus Groschner group [104,105]. A TRPC5 residue at a similar position, N584, was also implicated in regulating the TRPC5′s Ca^2+^ permeability by Chen et al. in 2017 [56], indicating for the first time that the uncharged, but polar residues like asparagine may also play a role in modulating the channel’s ability to carry Ca^2+^ cations through the pore of non-selective cation channels, weakly permeable to Ca^2+^. Indeed, mutating TRPC5-N584 to either aspartate or glycine significantly affected the relative Ca^2+^ permeability P_Ca_/P_Na_. TRPC5^N584D^ was about 5.7-fold more permeable to Ca^2+^ than that of TRPC5^N584G^ and 2-fold more permeable than that of the wild type human TRPC5 [56]. Notably, in all TRPC structures, the side chains of the residues homologous to E630 of hTRPC3 [104] and N584 of mTRPC5 [56] are facing the channel cation conduction pathway [97,99,101,102,103] (Figure 3). 

### 3.3. Disulfide Bond

**Figure 4:**
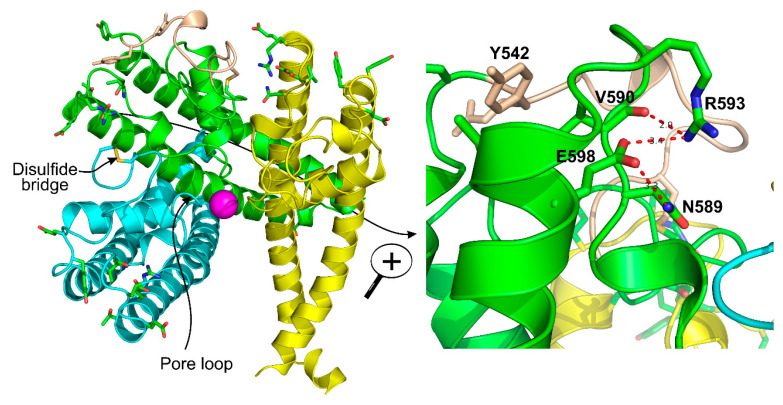
The structures of three out of four TRPC5 subunits are shown in the left panel. The position of the antigen for the E3 antibody which inhibits TRPC5 activity is shown in wheat color. This E3 antibody was developed by the Beech group [108]. The disulfide bridge is shown in yellow within the cyan subunit of TRPC5. Na^+^ in the cation conduction pathway of TRPC5 is depicted as a magenta sphere. The right panel shows a magnified view of the green subunit. The R593 residue which Chen et al. [56] named as “molecular fulcrum” is labeled, and its interactions with neighboring residues are shown using the red dotted lines. The Y542 residue that is involved in regulating Gd^3+^ sensitivity of TRPC5 is shown in wheat color. The mouse TRPC5 atomic coordinates were from PDB ID#: 5AEI.

A distinct property of TRPC4 and TRPC5 channels is the existence of a disulfide bridge within the E3 extracellular loop. The disulfide bridge is formed either between the extracellular cysteines C549 and C554 in mTRPC4 or C553 and C558 in mTRPC5 and is implicated in regulating the TRPC4 and TRPC5 function and gating [101,102,103,106] (Figure 4). The homologous cysteine residues are present in TRPC1 but not in TRPC3 or TRPC6. Xu et al. provided evidence that TRPC5 can be activated by reduction of the C553–C558 disulfide bridge with extracellular reduced thioredoxin or dithiothreitol and that C553A and C558A TRPC5 mutants consistently exhibit constitutive activity [106]. However, later studies demonstrated that the C553A, C553S, C558A, C558S, and C553A–C558A mutants of TRPC5 were not functional [103,107]. Hong et al. suggested that the C553–C558 disulfide bridge is critical for the stabilization of TRPC5 tetramers and the channel trafficking [107]. Indeed, the solved TRPC5 structure also suggests that the extracellular disulfide bridge stabilizes the channels’ extracellular domain and pore loop, and it may be important to gating in TRPC4 and TRPC5 [103]. Interestingly, Duan et al. demonstrated that the short sequence “TRAIDEPNN”, preceding the C553–C558 disulfide bridge of TRPC5, may be important for tuning the rate of inactivation of the channel [103]. However, further studies are still needed to clarify the physiological role of the C553–C558 disulfide bond in TRPC4 and TRPC5.

### 3.4. Cation Binding Sites

All of the identified TRPC structures revealed an intramembranous hydrophilic pocket on the cytoplasmic side in the vicinity of the S2 and S3 helices that serves as a cation binding site for Na^+^ and possibly Ca^2+^ [103]. This site is distinct from the cation conduction pathway. Sequence alignment revealed that the associated negatively charged Glu and Asp residues (E418, E421, and D439 in TRPC5) are highly conserved within the TRPC family [103]. It appears that the polar N436 residue also contributes to coordinating Na^+^ in the cation binding site [103]. Despite the fact that the cation binding site is identified in several TRPCs, its physiological role remains unknown at this time.

TRPC4 and TRPC5 uniquely contain an extracellular trivalent lanthanide binding site [41]. This extracellular cation binding site is located in the vicinity of the channels’ cation conduction pathway mouth [54]. The trivalent cations, such as La^3+^ and Gd^3+^, markedly potentiate the activity of TRPC4 and TRPC5 channels by acting at the extracellular cation binding site. However, both La^3+^ and Gd^3+^ potently blocked the TRPC3 and TRPC6 channel activity, probably via a pore block mechanism. The molecular determinants of the extracellular lanthanide binding site were delineated by Jung et al. [54] who demonstrated that the extracellular TRPC5′s Glu^543^, and Glu^595^ residues were critical for coordinating La^3+^. 

The reported Cryo-EM structure of TRPC5 [103] does not reveal any clear extracellular binding site that can coordinate La^3+^ or Gd^3+^ in TRPC5, although Glu^543^ and Glu^595^ residues are clearly seen at the end of the S5 helix and at the beginning of S6, respectively. This is consistent with the observation that lanthanides can potentiate only activated or spontaneously active TRPC5 [41,103], but not the resting TRPC5. Indeed, the TRPC5 structure depicts an inactive channel. Additionally, the lack of a clear extracellular binding site in the TRPC5 structure may be related to the fact that the structure was solved using the proteins purified in a divalent-free buffer [103]; the absence of Ca^2+^ may affect the channel conformation. 

Using computer modeling and molecular dynamics simulations, Chen et al. built a putative structure model of the TRPC5 pore region and the extracellular trivalent cation binding site [56]. The authors used the cryo-EM atomic coordinates of the open rat TRPV1 channel structure (PDB ID: 3J5Q) as a template, the only open TRP channel structure available at that time. TRPV1 is a lanthanide sensitive channel, justifying it as an optimal template. However, there are differences between the atomic structures of TRPC5 and TRPV1, therefore it may not be the best template for homology modeling of TRPC5. Despite this fact, the Chen TRPC5 model was useful to predict the role of two aromatic residues, Tyr^541^ and Tyr^542^, located just below the Glu^543^ residue at the base of the E3 in regulating trivalent cation-mediated effects in TRPC5. When these residues were mutated to alanine, the mutated channel lost its sensitivity to Gd^3+^ [56]. It is possible that the backbone-carbonyl oxygen atom of Tyr^542^ may contribute to coordinating a trivalent cation within the extracellular cation binding site. Only cryo-EM structures solved for TRPC5 proteins purified in the presence of Ca^2+^ and Gd^3+^ would be helpful for identifying the precise atomic arrangements within the extracellular cation binding site of TRPC5. 

### 3.5. Arg593 Serves as “Molecular Fulcrum” and Is Critical for GPCR-Gq-PLC-Dependent Gating of TRPC5

The homology model of TRPC5 described by Chen et al. [56] was also useful for identifying Arg^593^ as a critical residue determining the functional activity of TRPC5 and excluding a neighboring Lys^591^, which is also a positively charged residue. Chen et al. were first to report that Arg^593^ (Figure 4, right) is essential for GPCR-Gq-PLC-dependent gating of TRPC5 [56]. A TRPC5 mutant containing an alanine instead of arginine at the 593 position exhibited a reduced sensitivity to Gq-PLC activation [56]. On this basis, Chen et al. were first to propose that Arg^593^ may serve as a “molecular fulcrum” in the TRPC5 channel, possibly transmitting the gating effort force to the pore helix-loop unit [56]. 

The cryo-EM structure of TRPC5 confirmed that the Arg^593^ residue serves as a molecular fulcrum. This residue appears to form polar bonds with the Val^590^ carbonyl group and the carboxyl group of Glu^598^, possibly uncovering the molecular mechanism allowing the efficient transmission of gating force to TRPC5′s pore helix-loop. However, Arg^593^ is not conserved among other TRPC channels. There is a glycine in TRPC1, aspartate in TRPC3 and TRPC7, glutamine in TRPC4, or asparagine in TRPC6 at the position homologous to the 593 position of TRPC5. Therefore, neither the Chen et al. study nor the studies reporting the atomic structures of TRPC3, TRPC3, TRPC4, TRPC5, and TRPC6 revealed the common molecular mechanism underlying the GPCR-Gq-PLC-dependent gating of TRPC channels. 

Nevertheless, the solved TRPC channel structures provide an important structural basis for future investigations of the channel regulation and gating mechanisms. These efforts already now help facilitate the development of new drugs targeting TRPC channels. However, the fact that almost all of the reported high-resolution structures of TRPC channels were obtained in the channels’ closed conformations represents an obstacle because physiologically activated TRPC channels may exhibit different atomic arrangements in the critical regions of the proteins. Therefore, there is a need to direct the effort at solving the high-resolution atomic structures of the TRPC channels in open conformations. 

### 3.6. The Calmodulin Binding Site on TRPC4

**Figure 5:**
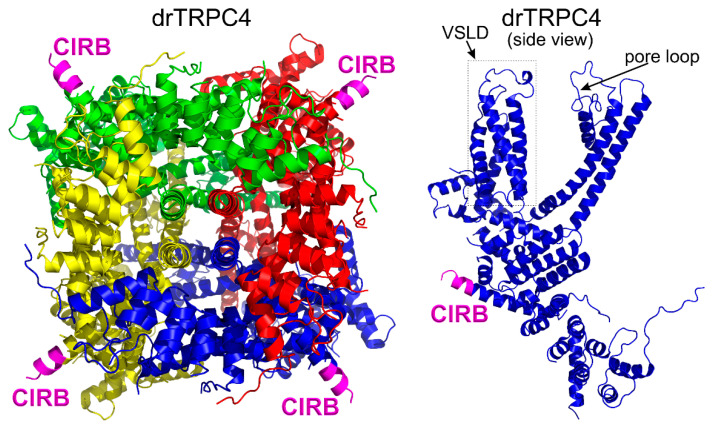
The atomic structure of apo *Danio rerio* TRPC4 (drTRPC4). VSLD stands for the voltage sensor-like domain: (**Left**) a view at the drTRPC4 protein from the cytosol; and (**Right**) a side view at drTRPC4. CIRB stands for the Ca^2+^-calmodulin-IP_3_ receptor binding domain. The CIRB domains are colored in magenta. The drTRPC4 atomic coordinate were from pdb ID: 6g1k [101].

The initial finding of the Mike Zhu group that Ca^2+^-calmodulin binds to and inhibits all TRPC channels has been recently confirmed by the yet unpublished data from the Raunser group (the preprint of their upcoming article is available for preview at the bioRxive Preprint Server for Biology [109]). The Raunser group structural data refined the position of the calmodulin binding site on *Danio rerio* TRPC4 to residues 688–703 (Figure 5). Initially, a slightly longer stretch of the CIRB domain (695–724 residues) on the mouse TRPC4’s C-terminus was identified using molecular biological and biochemical approaches by the Zhu laboratory [44]. The new TRPC4 structure data suggest that residues 704–725 are engaged in the *Danio rerio* TRPC4 protein core and should be inaccessible for calmodulin binding [109]. Vinayagam et al. reported that Ca^2+^-calmodulin binds to the tip of the rib helix (Figure 5) and that this results in the stabilization of the residues in and around the voltage-sensor-like domain which connects to the TRPC4 low gate, thus locking the gate [109].

## 4. Physiological and Pathological Functions of TRPCs Revealed Using Gene Knockout, Knockin and Pharmacological Approaches

### 4.1. Physiological Activators of TRPC Channels

**Figure 6:**
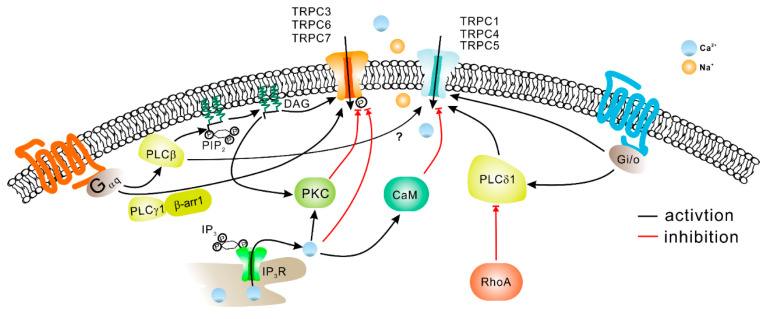
The mechanisms involved in regulating TRPC activity. Gα_q/11_-coupled-receptor activation leads to PLC-mediated hydrolysis of phosphatidylinositol 4,5-bisphosphate (PIP_2_) and production of inositol 1,4,5-trisphosphate (IP_3_) and diacylglycerols (DAG). IP_3_ activates IP_3_ receptors (IP_3_R) on the endoplasmic reticulum resulting in stored Ca^2+^ release and Ca^2+^ store depletion. DAG can directly activate TRPC3, TRPC6, and TRPC7 channels [40,123]. Additionally, DAG in conjunction with Ca^2+^ can activate PKC, which may in turn phosphorylate TRPC channels. Phosphorylation by PKC inhibits TRPC3/TRPC6 activity [120]. Ca^2+^ may decrease TRPC channel activity directly or via calmodulin (CaM). TRPC4 channel activity can be elicited not only downstream of Gα_q/11_, but also via Gα_i/o_ protein interaction. Besides DAG, TRPC3 and TRPC6 channels have been reported to be activated in PLCγ, IP_3_ or β-arrestin-1 dependent manner.

The activation of TRPC channels predominantly occurs downstream of the GPCR-G_q/11_-PLCβ and receptor tyrosine kinases coupled to PLCγ. It is well known that PLC stimulates the hydrolysis of PIP_2_ resulting in IP_3_ and DAG production as well as IP_3_-dependent Ca^2+^ release from the endoplasmic reticulum and consequent intracellular Ca^2+^ store depletion. Ca^2+^ itself is an important regulator of TRPC channels, potentiating TRPC5, but inhibiting TRPC3 and TRPC6 channels. This modulation is often mediated by calmodulin and other Ca^2+^-binding proteins. More detailed information about this kind of modulation can be found in the review article by Mike Zhu [110]. In addition to regulation by PLC and Ca^2+^, there are multiple other factors involved in the regulation of TRPC channel activity (Figure 6). It has been reported that Gq-PLCβ-DAG is not the only way to activate TRPC3. GPCR stimulation could recruit PLCγ and β-arrestin-1, assembled into a complex, to directly open TRPC3 channels in chromaffin cells and to promote the calcium-dependent acute catecholamine secretion [111] (Figure 6). Another group provided evidence that IP_3_ may activate the TRPC3 channel in an intracellular Ca^2+^ release independent manner [112]. On the other hand, PIP_2_ was reported by several groups to be essential for maintaining the activity of TRPCs channels [113,114,115]. However, the precise effect of PIP_2_ reduction on TRPC channel activity remains unclear. FRET was used to measure PIP_2_ or DAG dynamics concurrently with TRPC6/C7 current activation or inactivation after the stimulation of the PLC pathway. It was demonstrated that the activation of the channels correlated with the kinetics of PIP_2_ reduction and the inactivation was mediated by the dissociation of PIP_2_ [116]. 

Although TRPC channels are generally assumed to be activated by the Gq-coupled receptors-PLC pathway (Figure 6), it was reported that open TRPC4 may interact with Gα_i2_ but not with Gαq. Thus, it appears that the modulation of TRPC4 activity by Gα_i2_ can be mediated through the direct protein–protein interaction [117]. Activity of TRPC4 channels can be uniquely modulated through the small GTPase RhoA. Thakur et al. demonstrated that the TRPC4 activation required the combined contribution of Gα_i/o_ protein and PLCδ1. The PLCδ1-dependent activation of TRPC4 mediated by Gα_i/o_ was abolished by constitutively active RhoA, and this mechanism was not observed in TRPC5 [118]. Trans-activation response RNA-binding protein 2 (Tarbp2) was reported as a modulator for TRPC4 channels. Tarbp2 binding to the C terminus of TRPC4 induced the upregulation of the channel activity and increased the cytosolic Ca^2+^ concentration [119]. This resulted in a dynamic regulation of Dicer, a protein requiring Ca^2+^-dependent proteolytic activation. 

When activated by a GPCR agonist, TRPC6 exhibits robust activity that decays over time. It was shown that the current decay was associated with the phosphorylation of TRPC6 at the residues Ser^448^ and Ser^768/714^ [120] or with the phosphorylation of GPCR itself [121], which was induced by a DAG-dependent activation of PKC (Figure 6). The phosphorylation of TRPC6 also affects the channel protein expression at the cell membrane. The phosphorylation site at Ser^14^ of TRPC6 is the target of MAPKs and proline-directed kinases like cyclin-dependent kinase 5 (Cdk5). Phosphorylation of TRPC6 at Ser^14^ enhances membrane expression of TRPC6 [122]. Conversely, Chen et al. demonstrated that TRPC6 activity decay may also be related in part to inactivation of the GPCRs by PKC-dependent phosphorylation [121].

### 4.2. TRPC-Mediated Cellular Ca^2+^ Signaling

The main function of TRPC channels is to translate extracellular signals carried by agonists of GPCRs into Ca^2+^ and Na^+^ influx that may consequently cause cell depolarization. Generally, the free cytosolic Ca^2+^ is low in the resting cells (~100 nm), and most of it is either buffered by Ca^2+^-binding proteins or stored in the endoplasmic reticulum. GPCR-PLC activation induces a transient release of stored Ca^2+^ from the endoplasmic reticulum in an IP_3_-dependent manner (Figure 6) even in the absence of TRPC activation. However, the TRPC-mediated Ca^2+^ influx may significantly prolong GPCR-associated cytosolic Ca^2+^ increases [124]. Remarkably, intracellular Ca^2+^ is a universal second messenger that regulates important functions in almost every cell type [125], including neuronal synaptic transmission, insulin secretion, cell growth, gene transcription, and muscle contraction. 

Under normal physiological conditions, the concentration of natural agonists for a GPCR is low, and they are quickly degraded, resulting in a transient activation of TRPC channels. However, dysregulated TRPC channels may mediate a more sustained Ca^2+^ and Na^+^ influx and cell depolarization. In neurons and muscles, TRPC-associated cell depolarization may trigger the activity of voltage-gated Ca^2+^ channels (Ca_V_) amplifying the initial Ca^2+^ influx. Indeed, TRPC channels have been considered as potential players in the modulation of action potentials in excitable cell types. They could play a role in regulating neuronal firing patterns and cardiac excitation-contraction coupling [126,127]. However, the specific role of Ca^2+^ entry mediated by TRPC channels and the mechanisms of TRPC channel interplay with voltage-gated Ca^2+^ channels are still not fully elucidated.

### 4.3. Physiological and Pathophysiological Functions of TRPC Channels

**Figure 7:**
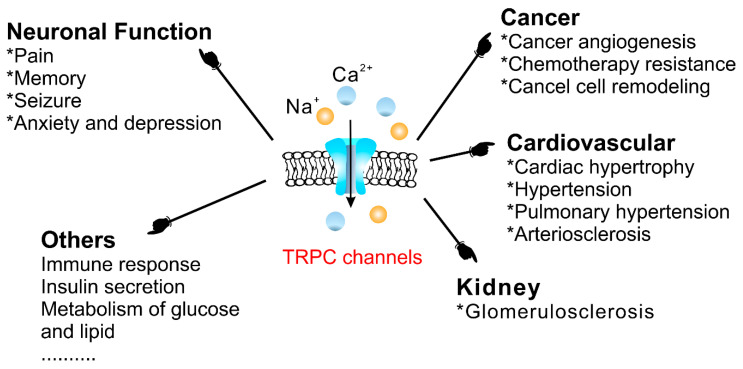
The physiological and pathophysiological roles of TRPC channels.

TRPCs are expressed in numerous mammalian cell types and are involved in many physiological and pathological processes. The gene-knockout (KO)/knock-in (KI) and pharmacological tools are main strategies to study TRPCs physiological and pathophysiological functions. These approaches continue to reveal multiple roles of TRPC channels in the cardiovascular system, skeletal muscle, pancreatic β-cells, neurons, bone, salivary gland cells, the immune system, and many other organ systems in mammals (Figure 7). Mice with genetically ablated TRPC channels are critical in initial evaluation of the roles of the channels. Small molecular modulators for TRPCs are another useful tool for the investigation of the physiological and pathophysiological roles of TRPCs. During the last two years, the development of new potent small molecule modulators of TRPC channels has been fueled by the recently solved TRPC structures, making evident progress. However, both approaches utilizing either gene-knockouts or small molecule modulators for studying TRPC function in vivo have limitations due to off-target effects of the drugs or the possibility of change in the expression of other genes in genetically modified mouse models. The wide distribution of TRPCs also creates an obstacle for investigating the channels’ specific functions in the in vivo setting using either genetic models or small molecule modulators because there may be a complex interplay between the changes in different organs on the systemic level which is difficult to decipher. Moreover, another problem is that TRPC channels may form heteromers not only within the TRPC subfamily, but also with the members of other TRP subfamilies. For example, TRPC1 was reported to form a functional heteromer with TRPV4 and TRPP2 channels in the vascular endothelium [128]. Thus, genetic ablation of TRPC1 basically creates a new heteromeric channel in the endothelium composed of TRPV4 and TRPP2 channels potentially exhibiting unique pharmacological and physiological properties. This may complicate the interpretation of TRPC1′s genetic deletion effects. Furthermore, small molecule modulators of TRPCs are often tested only in a limited set of heteromeric channels, such as TRPC1–TRPC5 heteromers, or heteromeric channels are not tested at all. Thus, there may be potentially a “TRPC channel escape” from small molecule modulator action in some tissues owing to the protection conferred by heteromerization. 

In this review, we discuss diseases and specific cell dysfunction associated with TRPC channel dysregulation uncovered using genetically modified mice and small molecule modulators.

#### 4.3.1. The Cardiovascular System

Cardiovascular disease remains the major cause of death in the world. TRPC KO mice and TRPC inhibitors were widely used to investigate the role of TRPC channels in the cardiovascular system, uncovering their contribution to cardiac hypertrophy, vascular tone regulation, pulmonary arterial hypertension (PAH), arteriosclerosis, and other pathophysiological conditions. 

In the heart, the phenomenon of hypertrophy results from increased cardiomyocyte size, which is secondary to pressure or mechanical overload. Several studies provided evidence that TRPC channels may contribute to the development of cardiac hypertrophy [129,130,131,132,133]. Hearts from hypertrophic patients had higher TRPC1 expression [134], and the cardiomyocytes from hypertrophic mouse models showed upregulated TRPC1/TRPC4 function [135]. Cardiac contractility was also associated with the TRPC3-mediated Ca^2+^ influx [136,137,138]. It was reported that TRPC channels do not always act alone. Eder et al. demonstrated that there are micro/nanodomains composed of TRPC3 and Na^+^/Ca^2+^ exchanger (NCX) in cardiomyocytes which are responsible for the increased cardiac contractility and susceptibility to the arrhythmogenic stimuli [139]. 

The gene-knockout evidence indeed revealed that the TRPC1 KO mice exhibit a reduced cardiac hypertrophy as compared to wild type (WT) mice when subjected to hemodynamic stress [134,140]. The TRPC3 KO mice also showed potential protection from pathologic cardiac hypertrophy [131]. Additionally, the data of knockdown (KD) of TRPC1 in cells suggested that the TRPC1-dependent mechanisms were associated with the calcineurin/nuclear factor of activated T cell (NFAT) or NF-κB signaling [140]. However, in a ATP-induced cardiomyocyte hypertrophy model, the activation of Ca^2+^-dependent NFAT signaling pathways were reported due to the upregulated TRPC3/6 channels [132]. Consistently, Seo et al. reported that the combined genetic deletion of TRPC3 and TRPC6 was protective against pressure overload, whereas neither TRPC3 nor TRPC6 individual gene genetic deletion did [130]. These results were validated with selective TRPC3/6 antagonists, GSK2332255B and GSK2833503A [141]. These inhibitors blocked pathological hypertrophic signaling in adult cardiac myocytes [130]. SK&F-96365, an unspecific inhibitor of receptor-operated, Cav, and TRPC channels, also attenuated cardiomyocyte hypertrophy induced by Ang II [133]. Interestingly, it was demonstrated that the protection conferred by TRPC3 deletion is not only due to the reduction of Ca^2+^ influx mediated by TRPC3, but also through the modulation of Ca_V_1.2 expression, which is downregulated in TRPC3 KO mice and leads to reduced response in phenylephrine-induced cardiac hypertrophy [131]. This indicates that while using KO approaches one should not overlook the contribution of expression changes of other genes.

Several TRPC channels have been implicated in modulating the physiological function of vascular smooth muscle and endothelial cells. In one of the pioneering works, the TRPC6 channel was demonstrated to play a key role in regulating myogenic tone [142], the vascular smooth muscle depolarizing response to intraluminal pressure increases. Later, it was shown that cerebral artery myogenic constriction may also involve the following signaling cascade: PLCγ1 → IP_3_Rs → TRPC6 → [Ca^2+^]i ↑ → TRPM4 [143]. Alvarez-Miguei et al. [144] reported that, although both TRPC3 and TRPC6 were expressed in mesenteric arteries and possibly formed heteromeric TRPC3/TRPC6 channels, only TRPC3 activity was associated with greater basal and agonist-induced contractions of mesenteric arteries from hypertensive animals compared to those isolated from normotensive control mice, implicating TRPC3 channels in the pathogenesis of essential hypertension. In this study, the authors used Pyr3/Pyr10 inhibitors to assess the contribution of TRPC3 and TRPC6 channels and found that increased TRPC3 expression favored depolarization of VSMCs [144]. Notably, the TRPC6 KO mice presented with hypertension and increased receptor-operated contractility due to upregulated expression of constitutively active TRPC3-type channels in the TRPC6 KO mouse vasculature [145]. Conversely, contractility of the aortic rings isolated from TRPC1 KO mice was similar to that observed in WT mice [146,147].

Contribution of TRPC channels to regulating the vascular tone may depend on other types of proteins involved in Ca^2+^ homeostasis. For example, in vascular smooth muscle cells (VSMCs), TRPC1 was shown to induce Ca^2+^ influx through interaction with Orai1 and Ca_V_1.2 channels [148]. In addition, Lemos et al. reported that Na^+^ influx through TRPC6 can drive Ca^2+^ influx through NCX working in the reverse mode in rat aorta smooth muscle cells [149]. Consistently, a cross talk between TRPC6 and NCX1 channels was reported in mesenteric artery smooth muscle cells from Milan hypertensive rats, indicating that those two dissimilar proteins are implicated in the hypertensive phenotype [150]. These data are in good agreement with the original reports indicating that TRPC3 channels can locally couple with NCX, with NCX working in the reverse mode to amplify Ca^2+^ influx [151]. Some of these studies utilized KB-R7943, an inhibitor of the NCX reverse mode, to further validate NCX contribution. However, one should be cautious while employing this NCX inhibitor when assessing TRPC function because KB-R7943 was reported to inhibit the activity of TRPC3, TRPC5 and TRPC6 channels [152]. 

Upregulated TRPC6 expression was detected in the smooth muscle layer of the coronary arteries isolated from metabolic syndrome pigs. Notably, the TRPC6 expression level positively correlated with the increased coronary artery contractility [153]. However, an in vitro study demonstrated that TRPC6 activity promoted phenotypic switching in vascular smooth muscle cells from the contractile phenotype to the highly proliferative “synthetic” phenotype through plasma membrane potential-dependent coupling with PTEN [154]. Conversely, inhibition of TRPC6 facilitated contractile differentiation of vascular smooth muscle cells [154]. The TRPC3 channel, a member of the same TRPC3/6/7 subgroup, was also found to facilitate phenotypical switch and proliferation of human smooth muscle cells from the coronary artery and aorta in vitro, and TRPC3 inhibition with Pyr3 decreased smooth vascular muscle proliferation [155]. Additionally, in vitro experiments provided evidence that TRPC1 inhibition decreases proliferation of cultured vascular smooth muscle cells [156]. Thus, it appears that the in vitro model cell systems may involve different mechanisms than those observed in in vivo models. 

As indicated above, TRPC3 was implicated in blood pressure regulation. Indeed, Liu et al. [157] found that aortas from spontaneously hypertensive rats (SHR) expressed higher levels of TRPC3 compared to control aortas from normotensive Wistar Kyoto rats. SHR aortas also exhibited a reduced angiotensin II-induced Ca^2+^ entry into vascular smooth muscle cells after TRPC3-knockdown using an siRNA approach; and increased Ca^2+^ entry was associated with higher blood pressure [157]. Additionally, TRPC3 was shown to play a role in maintaining mitochondrial Ca^2+^ homeostasis. Therefore, inhibition of TRPC3 led to decreased ROS and H_2_O_2_ production that is normally associated with increased activity of TRPC3 and TRPC6 channels [158]. Indeed, TRPC3 KO mice showed less severe hypertension through reduction of angiotensin II-induced mitochondrial ROS production [159], a finding that was validated using Pyr3 inhibitor. However, since Pyr3 blocks not only TRPC3 but also Orai1 channels [160], it is not yet clear whether TRPC3 channels are responsible alone for this effect. 

Hypoxic pulmonary vasoconstriction is a homeostatic mechanism in the pulmonary vasculature which occurs in response to alveolar hypoxia. It is meant to divert blood from hypoxic lung regions to those with normal oxygen content. Weissmann et al. found that deletion of TRPC6 eliminated acute hypoxic pulmonary vasoconstriction [161]. This suggests that TRPC6 may possibly serve as a therapeutic target for the maintenance of pulmonary hemodynamics and gas exchange under high altitude condition, and it also validates claims of TRPC6 contribution to the pathogenesis of hypoxic pulmonary vasoconstriction [161,162]. 

Pulmonary hypertension is a deadly disease, which is often associated with vascular remodeling. Initially, Malczyk et al. established that TRPC1 alone may underlie pulmonary vascular remodeling in response to hypoxia-induced pulmonary hypertension [163]. Later, Xia et al. reported that TRPC1–TRPC6 Double KO mice exhibited pulmonary hypotension and were better protected from chronic-hypoxia-induced pulmonary hypertension as compared to TRPC1 KO and TRPC6 KO mice [164], suggesting that the combined actions of TRPC1 and TRPC6 channels could exhibit larger influence on pulmonary artery remodeling under chronic hypoxia. Xia et al. also showed that, while TRPC1 KO mice were resistant to long-term hypoxia, TRPC6 KO mice were protected only from short-term (one-week) hypoxia, indicating that TRPC1 and TRPC6 differentially contribute to the pathogenesis of pulmonary hypertension. Xia et al. suggested that the simultaneous targeting of both TRPC1 and TRPC6 channels may be a possible therapeutic approach for treating pulmonary hypertension. However, they noted that other TRPC1/TRPC6-independent mechanisms may also contribute to increased pulmonary pressure under chronic hypoxia [164]. 

The endothelium is the innermost layer of vessels and is responsible for regulating the vascular tone. Dysfunctional endothelium contributes to initiating many vascular diseases including hypertension and atherosclerosis. Agonist-induced aortic vasorelaxation was markedly attenuated in TRPC4 KO mice [165]. Consistently, another study identified a missense SNP, TRPC4-I957V, associated with a reduced risk of myocardial infarction in diabetic patients, as a gain-of-function mutation resulting in a facilitated channel insertion into the plasma membrane, apparently promoting endothelium-dependent relaxation [166]. Additionally, TRPC1 was found to control endothelial permeability through an interaction with TRPV4 or TRPC4 in two studies utilizing endothelial cells from TRPC1 KO mice [167] or TRPC1/TRPC4 double knockout mice [168]. 

TRPC1 and TRPC4 were also found to have a large impact on endothelial repair function via regulating endothelial progenitor cell (EPC) growth cycle. Knockdown of TRPC1 or TRPC4 caused decreased store-operated Ca^2+^ entry and arrested the EPC cycle in G1. This supports the hypothesis that TRPC1 may regulate endothelial repair potential and could be a target for promoting vascular repair [169]. Yeon et al. investigated the contractility and relaxation of mouse mesenteric arteries from TRPC3-knockout mice and their results demonstrated that TRPC3 not only contributes to mediating vascular smooth muscle contractions, but also controls the release of NO from endothelial cells [170]. 

The involvement of TRPC6 in regulating the endothelial barrier function was investigated in the lungs of TRPC6 KO mice. TRPC6 KO mice were resistant to the lung injury induced by ischemia–reperfusion injury or endotoxins like LPS or histamine [171,172]. Silva et al. evaluated the effect of inhibitors for TRPC3/6 (Pyr3 and Pyr10) channels and TRPC4/5 (ML204) on endothelium-dependent relaxations in pre-constricted rat thoracic aortic rings [173]. Pyr3 caused a rapid reversal of acetylcholine relaxations, whereas the more selective TRPC3 blocker Pyr10 and TRPC4/5 blocker ML204 [174] had no effect. These data suggest that Ca^2+^ influx mediated by TRPC6 contributes to production and release of nitric oxide in endothelial cells and that upregulation of TRPC6 compromised the barrier function of the vascular endothelium. 

Traumatic brain injury is known to induce profound endothelial dysfunction in the systemic microcirculation [175]. Initially, the pathology was linked to arginase-1-dependent uncoupling of endothelial nitric oxide synthase. Chen et al. later reported that TRPC6 KO mice were also protected from traumatic brain injury-associated aortic endothelial dysfunction [176], implicating TRPC6 in the traumatic brain injury-induced pathology of systemic conduit circulation. In this study, the involvement of TRPC6 channel activation in mediating traumatic brain injury-induced aortic endothelial dysfunction was validated using a specific inhibitor of TRPC6 [176], larixyl acetate [177].

Arteriosclerosis is one of characteristics of metabolic syndrome and one of the major causes of coronary artery disease. Atherosclerosis is linked to vascular wall inflammation, and recruitment of T-cells and monocytes is implicated in the pathogenesis of the disease. Monocytes infiltrating the vascular wall transform into the macrophages that represent a major component of most atherosclerotic plaques, specifically in the coronary artery. Using an Ossabaw pig model of metabolic syndrome-induced atherosclerosis, Li et al. found that increased abundance of TRPC1 protein expression in atheroma macrophages correlated with the degree of atherosclerosis [153]. These data suggest that the migration of monocytes/macrophages into the arterial wall may be associated with the upregulated TRPC1 channel expression [153]. The Vazquez group used a mouse model of atherosclerosis and found that the deficiency of TRPC3 in bone marrow reduced the necrotic core of atherosclerotic plaques [178]. Consistently, the same group later reported that the macrophage-specific TRPC3 KO mice presented with reduced necrotic core and macrophage content in atherosclerotic plaques in ApoE^−/−^ mice [179]. On the contrary, ApoE*^−/−^* mice with transgenic overexpression of TRPC3 in vascular endothelial cells exhibited more advanced atherosclerotic lesions with increased macrophage content as compared to WT mice [180]. This work identified that genetically elevated TRPC3-mediated Ca^2+^ influx promoted atherosclerosis via increasing vascular cell adhesion molecule-1 and phospho-IkBα in the transgenic endothelium. However, later studies provided evidence that increased expression of TRPC6 due to a reduction in miR-26a in ApoE*^−/−^* mice fed an atherogenic diet was associated with more advanced atherosclerosis [181]. Consistently, lysophosphatidylcholine, which is abundant in atherosclerotic plaques, was shown to activate TRPC6, to induce TRPC6 membrane translocation, and to inhibit endothelial cell healing in vivo via a Src → Calmodulin → PI_3_ kinase → PIP_3_ → TRPC6 pathway [182]. Lysophosphatidylcholine activation of endothelial TRPC5 also contributed to impaired endothelial healing [183]. In agreement, inhibition of TRPC5 by isoliquiritigenin was associated with decreased atherosclerosis in ApoE*^−/−^* mice [184]. Thus, more research is needed to clarify which specific TRPC channels are most important during progression of atherosclerosis. 

Summarizing the above described effects of TRPC genetic deletion or inhibition in the relation to the cardiovascular system, it appears that the TRPC3/6 subgroup has a larger contribution in promoting cardiovascular disease. This is consistent with the channel’s distribution in the cardiovascular tissues. However, there is evidence suggesting that TRPC4 and TRPC5 channels expressed in the endothelial cells may also contribute to regulating the vascular tone. Specifically, TRPC4 was implicated in mediating endothelium-dependent vasorelaxation [165], whereas TRPC5 was shown to mediate endothelium-dependent constriction by promoting the activation of COX-2 and subsequent prostaglandin production, at least in the mouse carotid artery [185]. 

#### 4.3.2. Cancer

Cancer is the second major cause of mortality in the world. The cancer cells’ Ca^2+^ homeostasis dysregulation is well documented. The abnormal Ca^2+^ signaling is observed during tumor initiation, progression, metastasis, and angiogenesis. TRPC1/4/5 channels were proposed as targets for cancer treatment [186] because of the following: (1) TRPC4 and TRPC5 contribute to the cancer angiogenesis, which is the hallmark of cancer. The channels’ downregulation studies on a cell level suggested that these TRPC channels and other Ca^2+^ permeable channels may modulate the angiogenesis within tumors through the VEGF pathway [187]. (2) TRPC5 plays a role in the cancer chemotherapy resistance. Ma et al. first revealed that the upregulated TRPC5 channel promoted the expression of p-glycoprotein, resulting in the drug resistance in the MCF-7 breast cancer cell line [188]. This resistance might be transferred to other cancer cells through the vesicles containing tumor specific TRPC5 and the introduction of the channel into other cells [189]. In addition, TRPC5 knockdown upregulated chemotherapy sensitivity to temozolomide (TMZ), the first-choice chemotherapy agent against glioblastoma [190]. (3) TRPC1 channels also interact with other calcium channels and form complexes with TRPC4, TRPC5, or Orai1 while also playing a role in cancer cell remodeling [191,192,193]. For example, in human colon cancer, a complex interplay between TRPC1/Orai1 and STIM2 was identified, with TRPC1/Orai1 expression upregulation being associated with increased SOCE and Ca^2+^ store content, whereas STIM2 downregulation underlying Ca^2+^ store depletion and promoting apoptosis resistance [191]. 

TRPC3 and TRPC6 are also attractive candidate-targets for tumor therapy. TRPC3 is activated downstream of follicle stimulating hormone (FSH), which stimulated the proliferation and invasion of ovarian cancer cells. FSH upregulated the expression of TRPC3, facilitating the influx of Ca^2+^. Conversely, TRPC3 knockdown using an siRNA-mediated silencing approach led to decreased expression of survivin, HIF1-α, and VEGF [194]. A Ca^2+^-enriched diet was reported to increase the prostate cancer risk. The dietary vitamin D and downregulated TRPC6 prevented the Ca^2+^-enriched diet induced acceleration of the progression of prostate intraepithelial neoplasia [195]. Furthermore, in MCF-7 and MDA-MB-231 cells, TRPC6 could interact with Orai1 and Orai3. This promoted the cell proliferation, migration, and invasion [196]. The antagonist for TRPC6, pyrazolo[1,5-a]pyrimidine, showed some potential against human gastric cancer [197]. The pancreatic cancer is often associated with fibrosis that is caused by activated pancreatic stellate cells. The fibrosis creates a hypoxic region that causes an accelerated pancreatic stellate cell growth. Pancreatic stellate cells with genetically deleted TRPC6 exhibited a reduced migration than WT pancreatic stellate cells under hypoxic conditions. Thus, TRPC6 may be a major protein in the pathway of pancreatic stellate cell activation by hypoxia [198].

#### 4.3.3. Diabetes Mellitus

TRPC channels are implicated in regulating glucose-stimulated insulin secretion and skeletal muscle glucose uptake, however, it is not fully understood whether the channels contribute to the pathogenesis of diabetes. Xu et al. [199] demonstrated that TRPC1 phosphorylation by PKCα is involved in promoting insulin secretion in the INS-1E cell model. Conversely, TRPC1 knockout mice fed with a high fat diet exhibited lower fasting plasma glucose levels compared to wild type mice [200]. Interestingly, single nucleotide polymorphism, rs7638459, in the TRPC1 gene was identified as a risk factor for developing type 2 diabetes in a Chinese population [201].

Knockout of TRPC6 in type I diabetic mice promoted insulin resistance [202], and TRPC3 knockdown resulted in a decreased insulin-mediated glucose uptake in adult skeletal muscle cells [203]. Additionally, TRPC3 inhibition prevented the potentiation of glucose-stimulated insulin secretion induced by the stimulation of GPCR40-PLC/PKC in pancreatic β-cells [204]. However, the mechanisms underlying the channels’ protecting role from diabetes risk are still unknown.

TRPC6 may contribute to the pathogenesis of diabetic nephropathy, a common complication in subjects with diabetes. Wang et al. reported that global genetic ablation of TRPC6 increased glomerular injury in an Akita mouse model of type I diabetes [202], with Akita-TRPC6 KO mice exhibiting reduced tubular injury compared to Akita mice but increased mesangial expansion. However, in a model of streptozotocin (STZ)-treated Dahl Salt-sensitive (Dahl SS) rats, deletion of TRPC6 had a protective effect in diabetic kidney disease, protecting the podocytes but not the glomerulus as a whole [205]. The STZ rats exhibited increased albuminuria and glomerular injury, but no difference was observed between Dahl SS and SS-TRPC6 KO mice. The same group also reported that TRPC6 deletion also protected podocytes from H_2_O_2_ damage caused by NADPH oxidase 4 (NOX4). Because of this, TRPC6 or NOX4 could be a target to slow the development of diabetic kidney disease [206].

Diabetic retinopathy (DR) is another severe complication of diabetes, which is one of the causes of blindness worldwide. Sachdeva et al. used a mouse model lacking four TRPC channels, the TRPC1/4/5/6 KO mice and induced hyperglycemia with STZ treatment to determine how TRPC channels affect diabetic retinopathy. Methylglyoxal (MG), a major mediator of diabetic retinopathy was measured as well as Glyoxalase 1 (GLO1), a main MG detoxifying enzyme. MG was significantly lowered in tetra-TRPC KO mice compared to wild type mice, and the GLO1 activity and enzyme levels were also higher in retinal extracts from the KO mice compared to wild type mice. These findings suggest a higher resistance of TRPC1/4/5/6 KO mice to diabetic retinopathy [207].

Notably, by analyzing the RNA sequencing data, Marabita and Islam reported that TRPC1 is the only TRPC channel which is expressed in human pancreatic β-cells [208,209]. Therefore, the information related to the roles of TRPC3, TRPC4, TRPC5, and TRPC6 channels in animal pancreatic β-cell models may not be applicable to human β-cells.

#### 4.3.4. Neuronal Function

Many TRPC channels are highly expressed in both the central and peripheral nervous system. Depletion of TRPC1 was associated with neurodegeneration, spatial working memory and learning/adaptation deficits [210,211], striatal neuron apoptosis [212], and different types of pain [213]. TRPC1 is the most highly expressed TRPC channel in the ventral midbrain neural stem cells [214]. The downregulation of TRPC1 prevented midbrain dopaminergic (DA) neuron differentiation induced by thyroid hormones [214]. Recently, it was demonstrated that TRPC1 deletion led to significantly increased apoptosis in striatum with a concurrent decrease in both 14-3-3Z and dynamin-1 (D2-DA receptor binding), two apoptosis-related proteins. Using double/triple-labeling and confocal microscopy, Martinez-Galan et al. [211] analyzed the neuroanatomical distribution of TRPC1 in the rat neocortex. They detected that TRPC1 is present in cortical pyramidal cells and enriched in SST/reelin cells, mainly at supragranular layers. However, TRPC1’s functional role in these cells remains unknown.

It was demonstrated that TRPC3 is crucial for neural development and formation of neuronal networks. When TRPC3 is deleted from undifferentiated mouse embryonic stem cells (mESCs), the cells either underwent apoptosis or lost the potential across the mitochondrial membrane. The TRPC3 knockout also had effects on the pluripotency and repressed the neural differentiation by the inhibition of expression of markers for neuronal cell types. This points to TRPC3 being important in the survival, pluripotency, and neural differentiation of mESCs [215].

TRPC4 and TRPC5 proteins are expressed in various cells of the nervous system and the analysis of the KO animal models already uncovered several functions of channels in neurons. One function is the regulation of synaptic transmission at several neural networks. For example, GABA release from granule cells is reduced upon downregulation of TRPC1 and TRPC4, indicating that TRPC1 and TRPC4 essentially contribute to glutamate induced Ca^2+^ elevation in granule cells [216]. TRPC5 negatively regulated neurite extension in young rat hippocampal neurons [217], whereas TRPC5 KO mice exhibited less conditioned fear [218]. Remarkably, TRPC5 KO mice also exhibited the attenuated policarpine-induced seizures, and less seizure-induced hippocampus cell death [219]. The underlying mechanism probably involves glutamate acting at PLC-coupled Group I mGluR1 and mGluR5 receptors activating a non-selective cation current which was likely mediated by TRPC5 channels. HC-070 is a potent TRPC4 and TRPC5 antagonist. Treatment with this compound significantly attenuates the anxiogenic effect in mice [220].

TRPC6 knockdown resulted in mitochondrial elongation and facilitated dentate granule cell degeneration after status epilepticus. This could make TRPC6 an important target for neurological diseases that are in tandem with malfunctioning mitochondrial dynamics [221]. Conversely, TRPC1 has been identified as a promising target for treating Huntington’s Disease (HD). TRPC1 knockdown studies show that inhibition of TRPC1 leads to improved motor performance and rescued medium spiny neurons spines in vitro and in vivo [222]. TRPC channels are also involved in learning and memory. TRPC1 KO mice showed alterations in spatial working memory and fear conditioning when compared to wild type. This indicates that TRPC1 plays a role in synaptic plasticity and spatial working memory processes [223]. TRPC1 was also implicated in altering the extinction of spatial reference memory [224]. Loss of TRPC1, TRPC4, and TRPC5 impacts plasticity by decreasing basal-evoked secretion, reducing the pool size of available vesicles, and accelerating synaptic depression during high-frequency stimulation. This supports the hypothesis that TRPC channels are important in regulating synaptic plasticity [225]. TRPC4 is one of the most expressed TRPC subtypes in the mammalian corticolimbic brain, but despite this, TRPC4 KO rats exhibited normal learning patterns. However, the TRPC4 KO rats self-administered cocaine less than the wild type rats. It has been hypothesized that TRPC4 regulates basal dopamine excitability. Because of the lack of effect on learning but the decrease in apparent desire for cocaine administration, TRPC4 could be a successful target for treatment of dopamine disorders [226].

Notably, there are neural activities that do not depend on TRPCs. Egorov et al. used triple-KO mouse line lacking TRPC1, TRPC4, and TRPC5 and hepta-KO mouse line lacking all seven TRPC channels (TRPC1-7) and found that graded persistent activity did not depend on TRPC channels in entorhinal cortex neurons [227].

Brain ischemia is a cause of severe neurological disease and may results in significant mortality. Ca^2+^ overload is a big factor in cerebral ischemia–reperfusion (I/R) injury. There is growing evidence that astrocytes play a part in the pathophysiology of ischemia. Chen et al. used a TRPC3/6/7-KO mice model and showed that these three TRPC channels represent potential targets to alleviate brain damage after I/R injury. TRPC3/6/7 KO mice showed reduction in NF-kB (pro-apoptotic) and an increase in AKT (anti-apoptotic). Overall, this led to decreased brain damage following I/R injury via inhibition of astrocyte apoptosis [228]. Other studies revealed a decrease in I/R damage when the TRPC5 channel was inhibited or genetically deleted [229]. TRPC5 is also involved in the brain’s response to acute injury such as epilepsy, trauma, and stroke. The exposure of cultured cortical neurons to H_2_O_2_ leads to Zn^2+^-triggered Ca^2+^ influx, which may result in neuronal death [230]. Park et al. reported that H_2_O_2_–induced neuronal death in wild type cortical neuron cultures was significantly decreased in the presence of ML204, a TRPC4/TRPC5 inhibitor, implicating TRPC4 and TRPC5 channels. Consistently, mixed cortical neurons isolated from TRPC5 KO mice showed strong resistance to H_2_O_2_-induced death. Additionally, neuronal death was decreased by NU6027 (6-cyclohexylmethoxy-5-nitroso-2,4-diaminopyrimidine), a cyclin-dependent kinase (CDK) inhibitor that also potently inhibited TRPC5 activity in a CDK-independent manner [230]. TRPC1 has the opposite role in I/R cerebral injuries. Contrary to TRPC5 KO mice, TRPC1 KO mice exhibited a worsened brain infarction, edema, neurological severity score, memory impairment, neurological deficits, and oxidative stress. Oppositely, TRPC1 upregulation inhibited the increase in reactive oxygen species [231].

The expression of TRPC6 is lower than TRPC3 or TRPC7 in the central nervous system [232]. In cerebellar Purkinje cells, the TRPC3 channel is involved in motor coordination by modulation of mGluR-dependent synaptic transmission [233]. TRPC3 mediated slow excitatory postsynaptic currents (sEPSCs) in cerebellar Purkinje neurons that are triggered by mGluR1. The sEPSCs are then augmented by type B γ-aminobutyric acid receptors (GABA_B_Rs). However, in TRPC3 KO mice this augmentation is completely absent while in other TRPC KOs it remains intact. Thus, the coupling of mGluR1 and TRPC3 may have implications in essential cerebellar functions [234]. TRPC3 gene and protein expression in hippocampal neurons was increased in contextual fear memory deficits, demonstrating that TRPC3 regulates hippocampal neuron excitability associated with memory function [127]. Recently, a study showed that when TRPC3 was blocked by the channel inhibitor, the amplitude of inspiratory motoneuronal activity was significantly reduced. This indicates that TRPC3 plays fundamental roles in respiratory pattern regulation [235]. It is reported that TRPC7 contributes to the initiation of seizures. TRPC7 KO mice show interrupted acute severe seizures when stimulated by pilocarpine. Downregulated TRPC7 inhibited long-term potentiation at CA3 recurrent collateral synapses [236]. TRPC3 also has effects on pilocarpine-induced seizures. TRPC3 KO both reduces behavioral manifestations and the RMS power of the seizures. This suggests that TRPC3 could be an effective target for novel anticonvulsive drugs [237]. In TRPC1/4 double KO mice, the large depolarizing plateau potential that underlies the epileptiform burst firing was completely absent and was 74% eliminated in TRPC1 KO. This indicates that TRPC1/4 channels are important for mediating this plateau potential. Moreover, excitotoxic cell death was also mediated by these channels [238]. TRPC1 has been implicated in mechanosensitivity of dorsal root ganglion (DRG) neurons. Knockdown of TRPC1 showed a 65% reduction of neurons with stretch activated responses according to one study [72]. Thus, TRPC1 channels may contribute to mechanosensitivity of DRG neurons along with Piezo channels [239,240].

In the sensory neurons, TRPV1 and TRPA1 channels are already known to be implicated in the peripheral detection or transmission of nociceptive stimuli. In guinea-pig DRG neurons, the combined application of TRPA1 antagonist and TRPV1 antagonist had no effect on the Ca^2+^ signal induced by nitro-oleic acid (OA-NO2), which is known as a metabolic and anti-inflammatory signaling mediator contributing to the resolution of inflammation. However, BTP2, a broad spectrum TRPC antagonist, completely inhibited the response to OA-NO2. Therefore, the modulation of sensory neuron excitability via actions on multiple TRP channels might contribute to the anti-inflammatory effect of OA-NO2 [241]. However, due to poor selectivity of BTP2, no definite conclusions can be made about which specific TRPC channels plays the most important role in OA-NO2 effects. It is reported that TRPC1 and TRPC3 are the major TRPC subunits in rat DRGs, especially in small and medium diameter neurons. Using genetic and pharmacological tools, it was demonstrated that there is a link between activation of pro-inflammatory receptors and calcium homeostasis through TRPC3-containing channels which operate both in a receptor and store-operated manner [242]. TRPC5 channels may also contribute to nociceptive signaling because TRPC5 KO mice exhibit reduced nociceptive thresholds (thermal and mechanical) in a complete Freund’s adjuvant-induced unilateral arthritis model [243].

Using TRPV1/TRPM3/TRPA1 triple knockout (TKO) mice, Vandewauw et al. demonstrated that the initiation of the acute heat-evoked pain response in sensory nerve endings relies on three functionally redundant TRP channels [244]. Based on the expression pattern and function of TRPCs on DRGs, it is possible that TRPCs also contribute to the fault-tolerant mechanism to avoid injury. Some TRPC channels were implicated in touch and hearing sensations. TRPC3 and TRPC6 double KO animals lost the sensitivity to light touch and exhibited impaired hearing. However, this was not observed in any of the single KOs [245]. When the study was expanded to analyze deficits in global quadruple TRPC1, TRPC3, TRPC5, and TRPC6 null mutant mice and quadruple knockout mice, larger deficits in touch and hearing were observed when compared to the TRPC3/TRPC6 double knockouts.

#### 4.3.5. The Uterus

TRPC1, TRPC3, TRPC4, and TRPC6 proteins are expressed in both uterine myometrial smooth muscle (HMSM) cells and endometrial stromal cells [246,247]. Initially, TRPC1 was implicated in mediating Ca^2^⁺ entry during decidualization of human endometrial stromal cells [248]. Later, it was demonstrated that the stromal cells also express TRPC4 and TRPC6 besides TRPC1, and TRPC6 agonists induced Ca^2+^ increases in the uterine stromal cells [249]. Additionally, TRPC pathways play a significant role in mediating oxytocin-induced contractions in the myometrium of pregnant and non-pregnant animals [250]. Pyr3 (10 μm) inhibited oxytocin-induced contractions on uteri of non-pregnant and early pregnancy stage, indicating that there is a contribution of TRPC3 to uteri contraction [250]. Interestingly, TRPC3 function decreased with advancement of pregnancy, probably contributing to the quiescent state of the pregnant uterus. Conversely, in preterm labor patients, TRPC3 expression was significantly increased [251]. Consistently, TRPC3 KO significantly decreased the occurrence of preterm labor in mice [251].

Notably, Hasna et al. showed that TRPC6 may regulate the uptake of essential metal ions in the placenta during pregnancy. TRPC6 KO mice exhibited a reduced litter size, structural changes of the placenta, and altered mRNA levels of CD31 and Gcm1 (two important markers of placental development) compared to WT mice. Uniquely, TRPC6 KO mice had elevated levels of zinc in the placenta, liver, and kidney during development and elevated amounts of iron in the fully developed adult brain and liver [252].

#### 4.3.6. The Gastrointestinal Tract

TRPC channels have also emerged as important contributors to smooth muscle function regulation in the gastrointestinal tract [253]. In human and monkey colonic smooth muscle, basal activation of TRPC1, TRPC3, TRPC4, and TRPC7 were shown to contribute to modulating the resting membrane potential and play an important role in determining basal gut smooth muscle excitability [254]. Although neither solo-TRPC4 KO nor solo-TRPC6 KO was shown to affect the spontaneous contractile activity of intestine longitudinal strips, the genetic ablation of both TRPC4 and TRPC6 (double KO) significantly reduced muscarinic receptor induced depolarization and contractile responses [255]. Tsvilovskyy et al. concluded that TRPC4 and TRPC6 channel activation are critical for stimulating the muscarinic cation current (mICAT) in intestinal smooth muscle [255,256], indicating that the mICAT may consist of TRPC4 and TRPC6 and that their activation may be important for accelerating intestinal motility. It has been reported that the inhibitory effect of anesthetic agents, such as isoflurane, decreasing gastrointestinal tract motility is mediated by inhibiting the G-protein signaling that is important for stimulation of mICAT formed by TRPC4 and TRPC6 channels [257]. However, the reports on the function of TRPCs in the gastrointestinal tract are scarce and new investigations are needed to better understand their contribution to regulating gut function.

#### 4.3.7. The Kidneys

TRPC6 channels are expressed on podocytes in the kidneys. Fifteen years ago, Winn et al. and Reiser et al. almost simultaneously reported the existence of several human TRPC6 mutations (P112Q, R895C, and E897K) associated with focal segmental glomerulosclerosis (FSGS) [94,95,258], a familial autosomal dominant disease. The identified FSGS-linked TRPC6 mutations were gain-of-function mutations. Later, many additional mutations were identified (for example, M132T [259]). These TRPC6 gain-of-function mutants mediated an elevated Ca^2+^ influx leading to glomerulosclerosis [260,261,262]. However, about 25% of FSGS-linked mutations in the TRPC6 gene were loss-of-function mutations (N125S, L395A, G757D, L780P, and R895L), and some of them were identified in pediatric patients [263]. The involvement of loss-of-function TRPC6 mutations in the pathogenesis of FSGS indicates that, while elevated TRPC6-mediated Ca^2+^ influx in podocytes is detrimental for the kidney function, some basal level of TRPC6 activity may still be required for normal function of podocytes in the kidney glomeruli, especially in pediatric patients [264]. Notably, TRPC6 channels are not the only TRPC channels expressed in the kidney, and podocytes are not the only kidney cell expressing TRPC6. The measurements of single-channel activity in isolated glomeruli revealed that increased TRPC5 activity is also associated with proteinuric disease progression, whereas TRPC6 activity appears to be homeostatic [265]. That may explain the fact that the inhibition of TRPC5 delayed the progression of kidney disease. In TRPC6 knockdown studies, it was suggested that TRPC6 was linked to FSGS through calpain-1 activation and through Talin-1 loss. This presents calpain or TRPC6 inhibition as a possible treatment for FSGS or other podocytopathies [266,267]. TRPC6-dependent constitutive activation of calcineurin-NFAT pathway was reported to contribute to the progression of FSGS [268].

TRPC6 activity was found to regulate actin stress fiber formation and focal adhesions in podocytes in response to metabotropic glutamate receptor 1 activation via a RhoA/ROCK-dependent pathway [269]. Additionally, TRPC6 knockdown reduced the paracelluar permeability to BSA in podocyte cultures after puromucin aminonucleoside (PAN)-induced podocyte injury. This finding may also be related to the regulation of the podocyte cytoskeleton [270]. In a murine unilateral ureter obstruction (UUO) model, a study showed that knockout of TRPC6 decreased inflammatory cell infiltration and fibrosis in UUO kidneys from New Zealand obese mice. This validates the hypothesis that TRPC6 is involved in fibrosis and supports the view that TRPC6 expression is important in the development of progressive kidney disease. Thus, targeting TRPC6 may be a promising therapeutic strategy for renal fibrosis and immune cell infiltration in polygenic models for the human metabolic syndrome [271]. Studies with TRPC6 KO mice also showed that TRPC6-mediated Ca^2+^ influx plays a role in suppressing autophagy triggered by oxidative stress in renal proximal tubular cells. This makes TRPC6 a possible target for treatment of renal oxidative stress injury [272].

The roles of TRPC1, TRPC3, and TRPC4 channels, which are also expressed in the kidney, are less clear (for review, see [264]). Therefore, further research is needed to determine these three channels’ effects on kidney function.

#### 4.3.8. Others

The expression of TRPCs is also detected in myoblasts, myotubes, platelets, megakaryocytes, salivary glands, osteoblasts and osteoclasts, and even dental pulp cells. The cellular functions of TRPCs in those cells is based on their ability to mediate Ca^2+^ influx or cell depolarization, but in some cases, the channel’s role is still controversial or unknown.

Upregulation of TRPC3 and TRPC6 was associated with enhanced T lymphocytes apoptosis through the PLC-IP_3_ pathway in a model of sepsis [273]. In alveolar macrophages, TRPC6 was reported to contribute to shunting the transmembrane potential generated by proton pumping in low pH phagosomes, ensuring efficient pathogen clearance [274]. Additionally, increased accumulation of peritoneal leukocytes secreting inflammatory mediator was observed in TRPC5 KO versus WT, thioredoxin-treated and LPS-injected mice [275]. However, we are still far from understanding the full function of TRPCs in the immune system.

TRPC1 was reported to reduce exercise-dependent protection against high-fat diet-induced obesity and type II diabetes [200]. Krout et al. demonstrated that TRPC1 is the major Ca^2+^ channel in adipocytes and provided evidence that TRPC1 may promote increased autophagy and decreased apoptosis. These data reveal that TRPC1 may play a role in regulating adiposity.

In human blood platelets, the cytosolic free Ca^2+^ rises are essential for initiating both thrombosis and hemostasis. During combined thrombin and collagen stimulation of platelets, activation of the store-operated Orai1 channel [276] and TRPC3/TRPC6 channels followed by the reverse-mode operation of the Na^+^/Ca^2+^ exchanger is important for triggering cytosolic Ca^2+^ transients [277,278]. Recently, TRPC6 located on intracellular membranes was identified as a regulator for both the basal and passive Ca^2+^ leak rates from agonist-sensitive intracellular Ca^2+^ stores in resting platelets [279]. Thus far, it is unclear whether the role of TRPCs in platelet aggregation may be independent from Orai1 and STIM1.

Neuronal and pro-opiomelanocortin (Pomc)-specific loss of TRPC5 decreased energy expenditure and increased food intake resulting in elevated body weight, suggesting the link between TRPC5 expression in the brain and energy balance, feeding behavior, and glucose metabolism [280]. Rode et al. found that a conditional transgenic expression of dominant negative TRPC5 resulted in a significant reduction of body weight gain and reduced adipocyte size in ApoE*^−/−^* mice that were maintained on a Western diet for 6–12 weeks [281]. However, knockdown of TRPC1 led to weight gain and loss of metabolic control in mice [282]. Compared to wild type mice, TRPC5 KO mice exhibited a reduced cholic acid–induced enlargement of the liver. This was associated with a decrease of hepatic acid bile and lipid content in the TRPC5 KO mice, indicating that TRPC5 may be an important target for liver disease therapies [283].

It was reported that TRPC6 is implicated in specific allergic responses. Sel et al. found that TRPC6 KO mice exhibited reduced allergen-stimulated allergic responses compared to wild type mice which was evidenced by a decrease in levels of T-helper type 2 cytokines, such as IL-5 and IL-13. Conversely, loss of TRPC6 did not affect mucus production in the lungs [284]. In this study, agonist-induced contractility of trachea was increased in TRPC6 KO mice, probably due to upregulation of TRPC3.

TRPC1 and TRPC3 knockdown led to decreased viability in airway smooth muscle cells (ASMCs). The effect of these proteins on ASMCs comes partially through regulating Ca^2+^ influx [285]. Deletion of TRPC1 in mice prevents airway remodeling induced by house dust mite challenge. TRPC1 likely intensifies house dust mite-induced airway remodeling via epithelial-to-mesenchymal transition and STAT3/NF-κB signaling [286].

Table 1 summarizes the TRPC transgenic animal model data. Since the time when TRPC channels were cloned, a great amount of work has been done towards the understanding of pathophysiological and physiological functions of TRPC channels. However, the knowledge which has been obtained is still like a puzzle that is yet to be pieced together. More detailed information on TRPC’s distribution and TRPC’s-mediate Ca^2+^ signaling is needed to fully understand the big picture.

## 5. A Brief Guide to Small Molecular Modulators for TRPC Channels

There are several reviews summarizing the progress in the development of small-molecules agonists and antagonists of TRPC channels [290,291,292,293]. A brief overview of pharmacological tools available to investigate TRPC signaling is provided in Table 2 (agonists) and Table 3 (antagonists).

The first known modulator of TRPC activity was 1-oleoyl-2-acetyl-sn-glycerol (OAG), a cell permeable diacylglycerol analog initially identified as a direct agonist of TRPC3 and TRPC6 channels [40]. Later, pyrazole 3 (Pyr3, or Ethyl-1-(4-(2,3,3-trichloroacrylamide)phenyl)-5-(trifluoromethyl)-1H-pyrazole-4-carboxylate [294]) has become the first commercially available TRPC-subtype specific inhibitor. It has been later overshadowed by Pyr10 (*N*-[4-[3,5-Bis(trifluoromethyl)-1H-pyrazol-1-yl]phenyl]-4-methyl-benzenesulfonamide [160]), which is more selective for TRPC3 over store-operated channels. Pyr3 inhibited both TRPC3 and store-operated Orai1 with an IC_50_ value of 0.54 μm [160]. This compromised the Pyr3 ability to differentiate between Orai1 and TRPC3. In contrast, Pyr10 inhibits TRPC3 with an IC_50_ of 0.72 μm, while blocking Orai1 with a significantly higher IC_50_ of 13.08 μm [160].

ML204 has become the choice inhibitor for TRPC4 and TRPC5 channels (TRPC4: IC_50_ = 0.99 μm; and TRPC5: IC_50_ = 9.2 μm), displaying a 19-fold selectivity against receptor-coupled TRPC6 channel activation [295]. The discovery of TRPC4 and TRPC5 channel activator, the sesquiterpene (−)-englerin A has been another important milestone [296]. Importantly, this compound is relatively selective for TRPC4 and TRPC5 over other TRPC channels and acts extracellularly.

The development of inhibitors for any pharmacological target requires testing the off-target effects. The major problem of the TRPC field has been that in some studies, the TRPC modulators have been utilized without considering their target specificity. An example is 2-aminoethoxydiphenyl borate (2-APB), which was initially discovered as an inhibitor of IP_3_-induced Ca^2+^ release from intracellular Ca^2+^ stores with the IC_50_ of ~42 µm [297]. Later, it was shown that 2-APB potently modulates store-operated Ca^2+^ entry in a concentration dependent manner, enhancing SOCE at the concentrations of 1–10 μm and inhibiting at the concentrations of >50 μm [298] (for review, see [299]). However, soon after, it was discovered that 2-APB also inhibits 1-oleolyl-2-acetyl-sn-glycerol-dependent TRPC6 and menthol-dependent TRPM8 channel activity and induces TRPV1, TRPV2, and TRPV3 channel activity [300]. Finally, it has been reported that 2-APB potently inhibits TRPC5 channels [301]. Despite such a broad array of effects, 2-APB is still used as a specific inhibitor for each of those independent targets without scrutinizing the involvement of other off-target effects. Another example is SKF-96365 (1-[β-(3-(4-Methoxyphenyl)propoxy)-4-methoxyphenethyl]-1H-imidazole hydrochloride), which was initially reported as an inhibitor of voltage-gated Ca^2+^ influx and receptor-mediated Ca^2+^ entry without affecting Ca^2+^ release from the endoplasmic reticulum [302]. Later, however, it was shown to inhibit store-operated channels [299]. In fact, SKF-96365 is not a selective inhibitor of TRPC channels and can even inhibit voltage-gated T-type Ca^2+^ channels [303].

Although TRPC channels are the “classical”, and the first cloned TRP channels, thus far, the available small molecular modulators for the channels are not yet highly selective. Additional effort and investment are required to obtain more detailed information regarding their specificity and direct binding site capabilities. Fortunately, the availability of cryo-EM TRPC structures markedly facilitated the progress in drug discovery [304].

**Table 2:** Small molecule modulators for TRPC channels (Agonists).

Agonists	Chemical Structure	TRPC Channel (EC_50_/IC_50_)	Characteristics	Reference
GSK1702934A	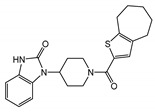	TRPC3 (0.08 μm) TRPC6 (0.44 μm)	No effect on TRPV4, TRPA1, TRPM1, TRPM4, CaV1.2, hERG, NaV1.5, or CXCR5 receptors at a concentration of 10 μmol/L	[305]
Pyrazolo-pyrimidines	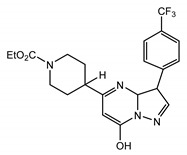	TRPC6 (0.89–6.28 µm) TRPC3 (0.02–0.45 μm)	Potency order: TRPC3 > TRPC7 > TRPC6	[306]
OptoDArG	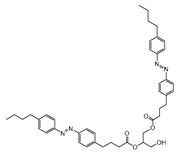	TRPC6 TRPC3 (30 μm was tested)	Photoswitchable DAG analogue containing two azobenzene photoswitchable moieties; active in cis-form at 365 nm and inactive at 430 nm	[50]
OptoBI-1	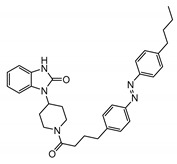	TRPC6 TRPC3 TRPC7 (10 and 30 μm were tested)	Photoswitchable azobenzene analogue of GSK1702934 A; active in the cis-form	[307]
PhoDAGs	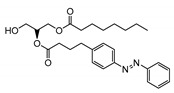	TRPC6 TRPC2 (5 and 50 μm were tested)	Photoswitchable DAG analogues; contain one photoswitchable moiety; active in cis-form at 370 nM and inactive at 460 nm	[308]
Hyp9	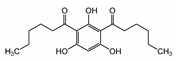	TRPC6 (1.26 μm)	A derivative of nonselective activator of TRPC3, TRPC6, and TRPC7	[305]
Artemisinin	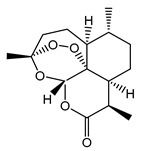	TRPC3 (33 µm)	May inhibit TRPC6	[309]
(−)-Englerin A	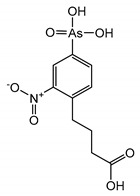	TRPC4 (11.2 nm) and TRPC5 (7.6 nm)	Selective activator	[296]

**Table 3:** Small molecule modulators for TRPC channels (Antagonists).

Antagonists	Chemical Structure	TRPC Channel (EC50/IC50)	Characteristics	Reference
Pyrazolo [1,5-a] pyrimidine (14a)	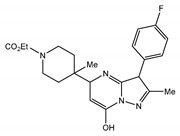	TRPC6 (1 μm)	Inhibits TRPC3/6/7 (TRPC6 > C7 > C3) with a very weak effect on TRPC4 and no effect on other TRP channels.	[197]
Pyrazole 3 (Pyr3)	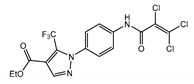	TRPC3 (0.5 μm) TRPC6 (> 10 μm)	Also inhibits STIM/Orai	[274]
Pyrazole 10 (Pyr10)	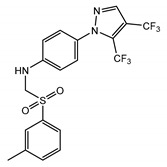	TRPC (0.72 μm) TRPC6 (> 10 μm)	More selective than Pyr3; does not inhibit STIM/Orai	[160]
BTDM	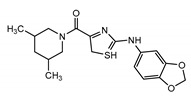	TRPC3 (0.01 μm) TRPC6 (0.01 μm)	The exact BTDM binding site in TRPC6 was defined by cryo-EM; wedges between the S5-S6 pore domain and voltage sensor-like domain to inhibit channel opening	[99]
GSK503A	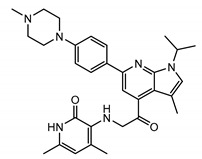	TRPC3 (0.003 μm) TRPC6 (0.021 μm)	Anilino thiazoles; good selectivity over other TRPA1,TRPV1, TRPV4, CaV1.2, hERG, and NaV1.5; in rodent models not orally bioavailable; high clearance, more suitable as in vitro tool	[141]
DS88790512	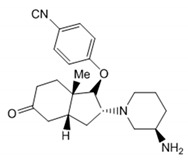	TRPC6 (0.011 μm)	Novel blocker of TRPC6; cyclohexanone derivative; excellent selectivity against hERG and hNaV1.5 channels	[310]
larixyl acetate	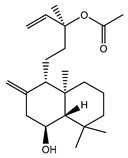	TRPC6 (0.1–0.6 μm)	Larch-derived labdane-type diterpenes; 12- and 5-fold selectivity compared with TRPC3 and TRPC7	[177]
BI749327	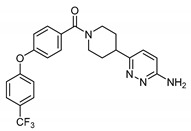	TRPC6 (13 nm)	BI 749327 is 85-fold more selective for mouse TRPC6 than TRPC3 (IC50 = 1100 nm) and 42-fold versus TRPC7	[311]
Pico145 (HC-608)	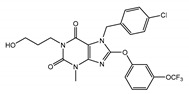	TRPC4 (63 pM) TRPC5 (1.3 nm)	Pico145 potency ranges from 9 to 1300 pM depending on the TRPC1/4/5 subtype while a range of other TRPC channels were unaffected	[312]
HC-070	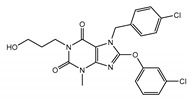	TRPC4 (46.0 ± 3.9 nm) TRPC5 (9.3 ± 0.9 nm)	HC-070 inhibits recombinant TRPC4 and TRPC5 homomultimers in heterologous expression systems with nanomolar potency	[220]
AC-1903	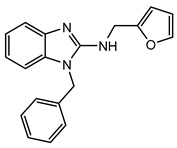	TRPC5 (14.7 μm)	AC1903 selectively blocks TRPC5 ion channels	[265]
ML204	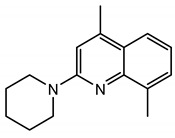	TRPC4 (0.99 μm) TRPC5 (9.2 μm)	ML204 exhibited modest inhibitory effects on TRPC6	[174]
Galangin	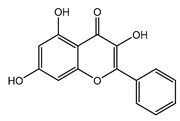	TRPC5 (0.45 μm)	Galangin is a natural product from the ginger family and a TRPC5 inhibitor depending on the substitution patterns of both the chromone core and the phenyl ring	[313]
SAR7334	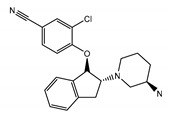	TRPC6 (7.9 nm)	SAR7334 inhibited TRPC3 and TRPC7-mediated Ca^2+^ influx into cells with IC50 s of 282 nm and 226 nm	[314]
SH045	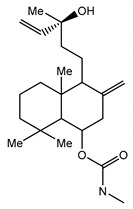	TRPC6(~5.8 nm)	IC50 for TRPC3 and TRPC7 are 0.84 μm and 0.22 μm, respectively	[315]
Bromoenol lactone (BEL)	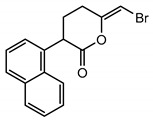	TRPC5 TRPC6 TRPC1–TRPC5 Cav1.2, SOCE	TRPC5: 10.6 μm TRPC6: 7.2 μm Cav1.2: 7.6 μm	[55]

## 6. Conclusions

Over the last 25 years of research in the TRPC field, it has been established that TRPC channels play numerous key physiological and pathophysiological roles. The structures of four TRPC channels were solved. The genetic knockouts of each of seven TRPC channels were constructed, including the hepto-TRPC1-7 KO mice lacking all seven TRPC channels. We know now that TRPCs are widely distributed throughout all of the major tissues and mediate Ca^2+^ and Na^+^ influx into the cells. Since Ca^2+^ is one of the most important intracellular second messengers, involved in the regulating of many critical process in human body starting from fertilization and ending only with the death of the cells, it is not surprising that the TRPC channels play important roles in many physiological and pathological mechanisms via regulating the cytosolic Ca^2+^ concentration and cell membrane potential. The usage of pharmacological tools and knock-out mice has been instrumental for the progress in the TRPC field. However, the drug specificity and possible TRPC gene deletion-associated alterations in the expression of other genes have to be considered in each individual case. Additionally, the possible contribution of other ion channels that may form heteromers with TRPCs should be evaluated while using the TRPC knockout models. In addition, the potential “TRPC channel escape” phenomenon associated with the TRPC channel subunit protection conferred by heteromerization with other channels should be taken into account during experiments utilizing pharmacological modulators targeting TRPC channels. In the last few years, the revolution in cryo-electron microscopy has enabled solving the high-resolution structures of TRPC channels. These advances enable the researchers to transition to a molecular level while aiming at understanding the channel function and its gating mechanisms. Although the precise molecular activation mechanisms are still unclear for most TRPC channels, the information on TRPC structures will definitely stimulate further advances. Despite a tremendous progress over the past 25 years, our understanding of TRPC channels in human health and diseases remains incomplete. Thus, while we celebrate the silver Anniversary of the discovery of TRPC channels, new research is still needed to answer the myriad unanswered questions.

## Figures and Tables

**Table 1:** The pathophysiological and physiological effects of TRPC knockouts and knockdowns.

Type	Effects	Authors	Reference
TRPC1 KO	TRPC1 KO mice exhibited increased mGluR agonist-induced non-selective inward currents in the CA1 neurons of the hippocampus	Kepura et al., 2020	[287]
	TRPC1 KO mice did not develop pulmonary hypertension under chronic hypoxia and had reduced vascular remodeling; however, right ventricular hypertrophy was similar to that in WT mice	Malczyk et al., 2013	[163]
	TRPC1 KO mice showed impairment in spatial working memory and fear memory formation but innate fear behavior was unaffected; activation of mGluR increased inward currents in hippocampal neurons from WT but not from TRPC KO mice	Lepannetier et al., 2018	[223]
	TRPC1 KO mice fed a high fat diet exhibited reduced fat mass, fasting glucose concentrations, and autophagy markers, whereas apoptosis markers were increased	Krout et al., 2017	[200]
	Tamoxifen-inducible TRPC1 genetic ablation in the hippocampus impaired mGluR-induced ERK1/2 activation, diminished mGluR-LTD, and decreased memory extinction	Yerna et al., 2020	[224]
	TRPC1 KO mice exhibited increased cerebral ischemia/reperfusion-induced infarction, neurological severity score, memory impairment, and oxidative stress.	Xu et al., 2018	[231]
	TRPC1 KO mice exhibited a reduced airway remodeling following house dust mite challenge with decreases in mucus production, cytokine secretion, and collagen deposition.	Pu et al., 2019	[286]
	TRPC1 KO mice exhibited no deficit of learning and memory under physiological conditions, but exacerbated learning and memory deficits induced by amyloid-β (Aβ), associated with Alzheimer’s disease	Li et al., 2018	[210]
	TRPC1 KO mice exhibited a reduced endothelial progenitor cell function secondary to calmodulin/endothelial nitric oxide synthase downregulation	Du et al., 2018	[167]
	Improved motor performance and an increased density of striatal medium spiny neuron spines in a mouse model of Huntington disease	Wu et al., 2018	[222]
TRPC3 KO	TRPC3 KO mice exhibited ameliorated hypertension through reduction of angiotensin II-induced mitochondrial ROS generation	Wang et al., 2017	[159]
	TRPC3 KO impaired the pluripotency of undifferentiated mouse embryonic stem cells and repressed their neural differentiation	Hao et al., 2018	[215]
	TRPC3 KO mice exhibited reduced pilocarpine-induced seizures and theta activity involved in status epilepticus	Phelan et al., 2017	[237]
	Phenylephrine-induced vasoconstriction and endothelium-dependent acetylcholine-induced vasodilation were reduced in TRPC3 KO mouse mesenteric arteries, whereas KCl- or pressure-induced vasoconstriction was unaltered.	Yeon et al., 2014	[170]
	Increase of mGluR1-induced slow EPSC by GABA_B_R activation was absent in TRPC3 KO mouse cerebellar Purkinje neurons but unaltered in TRPC1, TRPC4 and TRPC1/4/5/6 KO Purkinje neurons	Tian & Zhu, 2018	[234]
	TRPC3 KO mice exhibited a delayed occurrence of inflammation-induced preterm labor	Jing et al., 2018	[251]
TRPC3 KD	TRPC3 KD resulted in a decreased insulin-mediated glucose uptake in adult skeletal muscle cells	Lanner et al., 2009	[203]
	TRPC3 KD resulted in a reduced angiotensin II-induced Ca^2+^ influx in VSMC from spontaneously hypertensive rats	Liu et al., 2009	[157]
TRPC4 KO	TRPC4 KO mice exhibited a reduced self-administration of cocaine, but normal learning for natural reward	Klipec et al., 2016	[226]
	TRPC4 KO mice lack store-operated Ca^2+^ currents and agonist-dependent vasorelaxation	Freichel et al., 2001	[165]
TRPC5 KO	Compared with WT mice, TRPC5 KO mice had a reduced phosphatidylserine externalization and less apoptosis of cerebral neurons following ischemia-reperfusion	Guo et al., 2020	[229]
	Cortical neurons from TRPC5 KO mice were resistant to H_2_O_2_ toxicity as compared to WT neurons	Park et al., 2019	[230]
	Acetylchoine-induced endothelium-dependent contractions were smaller in TRPC5 KO carotid arteries compared to WT mice; TRPC5 contributed by stimulating COX-2-mediatedprostanoid production from carotid artery endothelial cells	Liang et al., 2019	[185]
	TRPC5 KO mice fed supplemental cholic acid had less liver enlargement than WT; hepatic bile acid was lower in TRPC5 KO mice	Alawi et al., 2017	[283]
	TRPC5 KO mice demonstrated reduced nociceptive thresholds (thermal and mechanical) in a complete Freund’s adjuvant-induced unilateral arthritis model	Alawi et al., 2017	[243]
	TRPC5 KO did not alter body temperature but was associated with increased accumulation of peritoneal leukocytes secreting inflammatory mediator in thioredoxin-treated LPS- injected mice.	Pereira et al., 2018	[275]
	Isoliquiritigenin from Glycyrrhiza glabra (Licorice) inhibits atherosclerosis by blocking TRPC5 channel expression and lipid serum levels in an apolipoprotein E (ApoE) knockout mouse model and angiotensin II-stimulated vascular smooth muscle cells (VSMCs)	Qi et al., 2020	[184]
	TRPC5 KO mice exhibited diminished innate fear levels in response to aversive stimuli and reduced response to metabotropic glutamate receptor activation in amygdala	Riccio et al., 2009	[218]
TRPC5 KD	Knockdown of TRPC5 increased chemosensitivity to temozolomide in glioblastoma	Zou et al., 2019	[190]
TRPC6 KO	Compared to Akita mice, TRPC6 KO Akita mice exhibited increased insulin resistance, mesangial expansion, and glomerular injury but reduced albuminuria and tubular injury	Wang et al., 2019	[202]
	In the unilateral ureter obstruction model, TRPC6 knockout was linked to decreased expression of pro-fibrotic, with pro-inflammatory genes being unaffected	Kong et al., 2019	[271]
	Reduces allergic airway response	Sel et al., 2008	[284]
	Induction of hypoventilation resulted in severe arterial hypoxemia not found in wild type	Weissmann et al., 2006	[161]
	Displayed reduced litter sizes and structural changes of the placenta	Hasna et al., 2019	[252]
	Protects mice from mTBI-induced aortic endothelial dysfunction;	Chen et al., 2019	[176]
	TRPC6 knockout has reno-protective effects in diabetic kidney disease, protecting the podocytes but not the glomerulus as a whole	Spires et al., 2018	[205]
	In TRPC6 KO mice, pancreatic stellate cells (PSCs) exhibited decrease migration as compared to WT PSCs under hypoxic conditions	Nielsen et al., 2017	[198]
	TRPC6 KO mice exhibited an elevated blood pressure and enhanced agonist-induced aortic contractility due to upregulation of constitutively active TRPC3-type channels	Dietrich et al., 2005	[145]
TRPC6 KD	Attenuated Ca^2+^ influx and the stress fiber formation induced by DHPG	Wang et al., 2019	[269]
	Prevented podocyte permeability increase induced by puromucin aminonucleaside	Sun et al., 2012	[270]
	Increased autophagic flux and mitigated oxidative stress-induced apoptosis of proximal tubular cells	Hou et al., 2018	[272]
	Decreased hypoxic increases of Ca^2+^ influx in PASMCs	Malczyk et al., 2017	[162]
	Increased TGF-β1-induced Akt phosphorylation at Ser473 in VSMCs	Numaga-Tomita et al., 2019	[154]
	Mitochondrial elongation, reduced phosphorylation of dynamin-related protein 1, and extracellular-signal-regulated kinase 1/2	Ko et al., 2017	[221]
	Unaltered ROS production, but increased inflammatory cytokines production compared to WT mice	Oda et al., 2017	[288]
TRPC6 KD	Increased density of striatal medium spiny neuron spines in a mouse model of Huntington disease	Wu et al., 2018	[222]
TRPC1 or TRPC3 KD	Asthmatic mouse airway smooth muscle cells exhibited upregulated TRPC1 and TRPC3 and increased proliferation. Knockdown either of the channels reduced proliferation.	Zhang et al., 2018	[285]
TRPC6 KO and TRPC5/6 DKO	TRPC6 KO mice and TRPC5/6 double KO mice were protected from H_2_O_2_-induced damage in a model of diabetic kidney disease	Ilatovskaya et al., 2018	[206]
TRPC1/4 KO TRPC5 KO TRPC1 KO	LTP and pilocarpine-induced seizures are reduced in TRPC5 KO mice, but are unaltered in TRPC1/4 KO mice; mGluR-mediated epileptiform bursting in the hippocampal CA1 area is not affected in TRPC5 KO mice, but is abolished in TRPC1 KO and TRPC1/4 DKO mice; seizure-induced neural cell death in the hippocampus was reduced in both TRPC5 KO and TRPC1/4 DKO mice	Phelan et al., 2013	[219]
TRPC1/4/5 KO	TRPC1/4/5 KO mice exhibited deficits in spatial working memory and relearning task, while spatial reference memory was not affected	Bröker-Lai et al., 2017	[126]
	TRPC1/4/5 KO mice exhibited decreased basal-evoked secretion, reduces readily releasable vesicles pool size, and accelerated synaptic depression during high-frequency stimulation, whereas TRPC5 KI showed short-term enhancement of synaptic strength during high frequency stimulation	Schwarz et al., 2019	[225]
TRPC3/6/7 KO	Compared to WT mice, triple TRPC3/6/7 KO mice had smaller infarct size when subjected to cerebral ischemia-reperfusion	Chen et al., 2017	[228]
TRPC1/4/5/6 KO	Tetra-TRPC KO mice are protected from hyperglycemia-evoked vasoregression. In addition, the KO mice are resistant to the STZ-induced reduction in retinal layer thickness	Sachdeva et al., 2018	[207]
TRPC1/4/5 or hepta-TRPC-KO	No change in persistent activity in entorhinal cortex neurons of layer V	Egorov et al., 2019	[227]
Hepta- TRPC KO	Thapsigargin-induced store-operated Ca^2+^ entry was absent in Orai1 KO anterior pituitary cells, but was unaffected in hepta TRPC KO; conversely, spontaneous intracellular Ca^2+^ oscillations related to electrical activity was reduced in hepta TRPC1-7 KO mouse cells and unaffected in Orai1 KO mouse cells	Núñez et al., 2019	[289]

## References

[1] Minke B., Wu C., Pak W.L. (1975). Induction of photoreceptor voltage noise in the dark in Drosophila mutant. Nature.

[2] Cosens D.J., Manning A. (1969). Abnormal electroretinogram from a Drosophila mutant. Nature.

[3] Wong F., Hokanson K.M., Chang L.T. (1985). Molecular basis of an inherited retinal defect in Drosophila. Invest. Ophthalmol. Vis. Sci.

[4] Montell C., Rubin G.M. (1989). Molecular characterization of the Drosophila trp locus: A putative integral membrane protein required for phototransduction. Neuron.

[5] Wong F., Schaefer E.L., Roop B.C., LaMendola J.N., Johnson-Seaton D., Shao D. (1989). Proper function of the Drosophila trp gene product during pupal development is important for normal visual transduction in the adult. Neuron.

[6] Hardie R.C., Minke B. (1992). The trp gene is essential for a light-activated Ca^2+^ channel in Drosophila photoreceptors. Neuron.

[7] Phillips A.M., Bull A., Kelly L.E. (1992). Identification of a Drosophila gene encoding a calmodulin-binding protein with homology to the trp phototransduction gene. Neuron.

[8] Niemeyer B.A., Suzuki E., Scott K., Jalink K., Zuker C.S. (1996). The Drosophila light-activated conductance is composed of the two channels TRP and TRPL. Cell.

[9] Reuss H., Mojet M.H., Chyb S., Hardie R.C. (1997). In vivo analysis of the drosophila light-sensitive channels, TRP and TRPL. Neuron.

[10] Obukhov A.G., Harteneck C., Zobel A., Harhammer R., Kalkbrenner F., Leopoldt D., Luckhoff A., Nurnberg B., Schultz G. (1996). Direct activation of trpl cation channels by G alpha11 subunits. EMBO J..

[11] Obukhov A.G., Schultz G., Luckhoff A. (1998). Regulation of heterologously expressed transient receptor potential-like channels by calcium ions. Neuroscience.

[12] Liu C.H., Wang T., Postma M., Obukhov A.G., Montell C., Hardie R.C. (2007). In vivo identification and manipulation of the Ca2+ selectivity filter in the Drosophila transient receptor potential channel. J. Neurosci..

[13] Hardie R.C. (2011). A brief history of trp: Commentary and personal perspective. Pflug. Arch..

[14] Inoue H., Yoshioka T., Hotta Y. (1985). A genetic study of inositol trisphosphate involvement in phototransduction using Drosophila mutants. Biochem. Biophys. Res. Commun..

[15] Devary O., Heichal O., Blumenfeld A., Cassel D., Suss E., Barash S., Rubinstein C.T., Minke B., Selinger Z. (1987). Coupling of photoexcited rhodopsin to inositol phospholipid hydrolysis in fly photoreceptors. Proc. Natl. Acad. Sci. USA.

[16] Putney J.W. (1986). A model for receptor-regulated calcium entry. Cell Calcium.

[17] Putney J.W. (2018). Forms and functions of store-operated calcium entry mediators, STIM and Orai. Adv. Biol. Regul..

[18] Minke B., Selinger Z. (1992). The inositol-lipid pathway is necessary for light excitation in fly photoreceptors. Soc. Gen. Physiol. Ser..

[19] Pollock J.A., Assaf A., Peretz A., Nichols C.D., Mojet M.H., Hardie R.C., Minke B. (1995). TRP, a protein essential for inositide-mediated Ca^2+^ influx is localized adjacent to the calcium stores in Drosophila photoreceptors. J. Neurosci..

[20] Hardie R.C. (1996). Excitation of Drosophila photoreceptors by BAPTA and ionomycin: Evidence for capacitative Ca^2+^ entry?. Cell Calcium.

[21] Scott K., Sun Y., Beckingham K., Zuker C.S. (1997). Calmodulin regulation of Drosophila light-activated channels and receptor function mediates termination of the light response in vivo. Cell.

[22] Hu Y., Vaca L., Zhu X., Birnbaumer L., Kunze D.L., Schilling W.P. (1994). Appearance of a novel Ca^2+^ influx pathway in Sf9 insect cells following expression of the transient receptor potential-like (trpl) protein of Drosophila. Biochem. Biophys. Res. Commun..

[23] Vaca L., Sinkins W.G., Hu Y., Kunze D.L., Schilling W.P. (1994). Activation of recombinant trp by thapsigargin in Sf9 insect cells. Am. J. Physiol..

[24] Hu Y., Schilling W.P. (1995). Receptor-mediated activation of recombinant Trpl expressed in Sf9 insect cells. Biochem. J..

[25] Harteneck C., Obukhov A.G., Zobel A., Kalkbrenner F., Schultz G. (1995). The Drosophila cation channel trpl expressed in insect Sf9 cells is stimulated by agonists of G-protein-coupled receptors. FEBS Lett..

[26] Gillo B., Chorna I., Cohen H., Cook B., Manistersky I., Chorev M., Arnon A., Pollock J.A., Selinger Z., Minke B. (1996). Coexpression of Drosophila TRP and TRP-like proteins in Xenopus oocytes reconstitutes capacitative Ca^2^+ entry. Proc. Natl. Acad. Sci. USA.

[27] Xu X.Z., Li H.S., Guggino W.B., Montell C. (1997). Coassembly of TRP and TRPL produces a distinct store-operated conductance. Cell.

[28] Wes P.D., Chevesich J., Jeromin A., Rosenberg C., Stetten G., Montell C. (1995). TRPC1, a human homolog of a Drosophila store-operated channel. Proc. Natl. Acad. Sci. USA.

[29] Zhu X., Chu P.B., Peyton M., Birnbaumer L. (1995). Molecular cloning of a widely expressed human homologue for the Drosophila trp gene. FEBS Lett..

[30] Zhu X., Jiang M., Peyton M., Boulay G., Hurst R., Stefani E., Birnbaumer L. (1996). trp, a novel mammalian gene family essential for agonist-activated capacitative Ca2+ entry. Cell.

[31] Vannier B., Peyton M., Boulay G., Brown D., Qin N., Jiang M., Zhu X., Birnbaumer L. (1999). Mouse trp2, the homologue of the human trpc2 pseudogene, encodes mTrp2, a store depletion-activated capacitative Ca^2+^ entry channel. Proc. Natl. Acad. Sci. USA.

[32] Philipp S., Cavalie A., Freichel M., Wissenbach U., Zimmer S., Trost C., Marquart A., Murakami M., Flockerzi V. (1996). A mammalian capacitative calcium entry channel homologous to Drosophila TRP and TRPL. EMBO J..

[33] Philipp S., Hambrecht J., Braslavski L., Schroth G., Freichel M., Murakami M., Cavalie A., Flockerzi V. (1998). A novel capacitative calcium entry channel expressed in excitable cells. EMBO J..

[34] Okada T., Shimizu S., Wakamori M., Maeda A., Kurosaki T., Takada N., Imoto K., Mori Y. (1998). Molecular cloning and functional characterization of a novel receptor-activated TRP Ca^2+^ channel from mouse brain. J. Biol. Chem..

[35] Boulay G., Zhu X., Peyton M., Jiang M., Hurst R., Stefani E., Birnbaumer L. (1997). Cloning and expression of a novel mammalian homolog of Drosophila transient receptor potential (Trp) involved in calcium entry secondary to activation of receptors coupled by the Gq class of G protein. J. Biol. Chem..

[36] Okada T., Inoue R., Yamazaki K., Maeda A., Kurosaki T., Yamakuni T., Tanaka I., Shimizu S., Ikenaka K., Imoto K. (1999). Molecular and functional characterization of a novel mouse transient receptor potential protein homologue TRP7. Ca(2+)-permeable cation channel that is constitutively activated and enhanced by stimulation of G protein-coupled receptor. J. Biol. Chem..

[37] Riccio A., Mattei C., Kelsell R.E., Medhurst A.D., Calver A.R., Randall A.D., Davis J.B., Benham C.D., Pangalos M.N. (2002). Cloning and functional expression of human short TRP7, a candidate protein for store-operated Ca^2+^ influx. J. Biol. Chem..

[38] Zitt C., Zobel A., Obukhov A.G., Harteneck C., Kalkbrenner F., Luckhoff A., Schultz G. (1996). Cloning and functional expression of a human Ca^2+^-permeable cation channel activated by calcium store depletion. Neuron.

[39] Zitt C., Obukhov A.G., Strubing C., Zobel A., Kalkbrenner F., Luckhoff A., Schultz G. (1997). Expression of TRPC3 in Chinese hamster ovary cells results in calcium-activated cation currents not related to store depletion. J. Cell Biol..

[40] Hofmann T., Obukhov A.G., Schaefer M., Harteneck C., Gudermann T., Schultz G. (1999). Direct activation of human TRPC6 and TRPC3 channels by diacylglycerol. Nature.

[41] Schaefer M., Plant T.D., Obukhov A.G., Hofmann T., Gudermann T., Schultz G. (2000). Receptor-mediated regulation of the nonselective cation channels TRPC4 and TRPC5. J. Biol. Chem..

[42] Lof C., Viitanen T., Sukumaran P., Tornquist K. (2011). TRPC2: Of mice but not men. Adv. Exp. Med. Biol..

[43] Kiselyov K., Xu X., Mozhayeva G., Kuo T., Pessah I., Mignery G., Zhu X., Birnbaumer L., Muallem S. (1998). Functional interaction between InsP3 receptors and store-operated Htrp3 channels. Nature.

[44] Tang J., Lin Y., Zhang Z., Tikunova S., Birnbaumer L., Zhu M.X. (2001). Identification of common binding sites for calmodulin and inositol 1,4,5-trisphosphate receptors on the carboxyl termini of trp channels. J. Biol. Chem..

[45] Zhang Z., Tang J., Tikunova S., Johnson J.D., Chen Z., Qin N., Dietrich A., Stefani E., Birnbaumer L., Zhu M.X. (2001). Activation of Trp3 by inositol 1,4,5-trisphosphate receptors through displacement of inhibitory calmodulin from a common binding domain. Proc. Natl. Acad. Sci. USA.

[46] Trebak M., St J.B.G., McKay R.R., Birnbaumer L., Putney J.W. (2003). Signaling mechanism for receptor-activated canonical transient receptor potential 3 (TRPC3) channels. J. Biol. Chem..

[47] Venkatachalam K., Zheng F., Gill D.L. (2003). Regulation of canonical transient receptor potential (TRPC) channel function by diacylglycerol and protein kinase C. J. Biol. Chem..

[48] Lemonnier L., Trebak M., Putney J.W. (2008). Complex regulation of the TRPC3, 6 and 7 channel subfamily by diacylglycerol and phosphatidylinositol-4,5-bisphosphate. Cell Calcium.

[49] Trebak M., Lemonnier L., DeHaven W.I., Wedel B.J., Bird G.S., Putney J.W. (2009). Complex functions of phosphatidylinositol 4,5-bisphosphate in regulation of TRPC5 cation channels. Pflug. Arch..

[50] Lichtenegger M., Tiapko O., Svobodova B., Stockner T., Glasnov T.N., Schreibmayer W., Platzer D., de la Cruz G.G., Krenn S., Schober R. (2018). An optically controlled probe identifies lipid-gating fenestrations within the TRPC3 channel. Nat. Chem. Biol..

[51] Tang Y., Tang J., Chen Z., Trost C., Flockerzi V., Li M., Ramesh V., Zhu M.X. (2000). Association of mammalian trp4 and phospholipase C isozymes with a PDZ domain-containing protein, NHERF. J. Biol. Chem..

[52] Obukhov A.G., Nowycky M.C. (2004). TRPC5 activation kinetics are modulated by the scaffolding protein ezrin/radixin/moesin-binding phosphoprotein-50 (EBP50). J. Cell Physiol..

[53] Storch U., Forst A.L., Pardatscher F., Erdogmus S., Philipp M., Gregoritza M., Mederos Y.S.M., Gudermann T. (2017). Dynamic NHERF interaction with TRPC4/5 proteins is required for channel gating by diacylglycerol. Proc. Natl. Acad. Sci. USA.

[54] Jung S., Muhle A., Schaefer M., Strotmann R., Schultz G., Plant T.D. (2003). Lanthanides potentiate TRPC5 currents by an action at extracellular sites close to the pore mouth. J. Biol. Chem..

[55] Chakraborty S., Berwick Z.C., Bartlett P.J., Kumar S., Thomas A.P., Sturek M., Tune J.D., Obukhov A.G. (2011). Bromoenol lactone inhibits voltage-gated Ca2+ and transient receptor potential canonical channels. J. Pharmacol. Exp. Ther..

[56] Chen X., Li W., Riley A.M., Soliman M., Chakraborty S., Stamatkin C.W., Obukhov A.G. (2017). Molecular Determinants of the Sensitivity to Gq/11-Phospholipase C-dependent Gating, Gd^3+^ Potentiation, and Ca^2+^ Permeability in the Transient Receptor Potential Canonical Type 5 (TRPC5) Channel. J. Biol. Chem..

[57] Zeng F., Xu S.Z., Jackson P.K., McHugh D., Kumar B., Fountain S.J., Beech D.J. (2004). Human TRPC5 channel activated by a multiplicity of signals in a single cell. J. Physiol..

[58] Subedi K.P., Ong H.L., Ambudkar I.S. (2017). Assembly of ER-PM Junctions: A Critical Determinant in the Regulation of SOCE and TRPC1. Adv. Exp. Med. Biol..

[59] Liao Y., Erxleben C., Yildirim E., Abramowitz J., Armstrong D.L., Birnbaumer L. (2007). Orai proteins interact with TRPC channels and confer responsiveness to store depletion. Proc. Natl. Acad. Sci. USA.

[60] Hong J.H., Li Q., Kim M.S., Shin D.M., Feske S., Birnbaumer L., Cheng K.T., Ambudkar I.S., Muallem S. (2011). Polarized but differential localization and recruitment of STIM1, Orai1 and TRPC channels in secretory cells. Traffic.

[61] So I., Chae M.R., Kim S.J., Lee S.W. (2005). Lysophosphatidylcholine, a component of atherogenic lipoproteins, induces the change of calcium mobilization via TRPC ion channels in cultured human corporal smooth muscle cells. Int. J. Impot. Res..

[62] Flemming P.K., Dedman A.M., Xu S.Z., Li J., Zeng F., Naylor J., Benham C.D., Bateson A.N., Muraki K., Beech D.J. (2006). Sensing of lysophospholipids by TRPC5 calcium channel. J. Biol. Chem..

[63] Xu S.Z., Muraki K., Zeng F., Li J., Sukumar P., Shah S., Dedman A.M., Flemming P.K., McHugh D., Naylor J. (2006). A sphingosine-1-phosphate-activated calcium channel controlling vascular smooth muscle cell motility. Circ. Res..

[64] Gomis A., Soriano S., Belmonte C., Viana F. (2008). Hypoosmotic- and pressure-induced membrane stretch activate TRPC5 channels. J. Physiol..

[65] Graham S., Ding M., Ding Y., Sours-Brothers S., Luchowski R., Gryczynski Z., Yorio T., Ma H., Ma R. (2010). Canonical transient receptor potential 6 (TRPC6), a redox-regulated cation channel. J. Biol. Chem..

[66] Spassova M.A., Hewavitharana T., Xu W., Soboloff J., Gill D.L. (2006). A common mechanism underlies stretch activation and receptor activation of TRPC6 channels. Proc. Natl. Acad. Sci. USA.

[67] McMahon H.T., Boucrot E. (2015). Membrane curvature at a glance. J. Cell Sci..

[68] Hirama T., Lu S.M., Kay J.G., Maekawa M., Kozlov M.M., Grinstein S., Fairn G.D. (2017). Membrane curvature induced by proximity of anionic phospholipids can initiate endocytosis. Nat. Commun..

[69] Maroto R., Raso A., Wood T.G., Kurosky A., Martinac B., Hamill O.P. (2005). TRPC1 forms the stretch-activated cation channel in vertebrate cells. Nat. Cell Biol..

[70] Dietrich A., Kalwa H., Storch U., Mederos y Schnitzler M., Salanova B., Pinkenburg O., Dubrovska G., Essin K., Gollasch M., Birnbaumer L. (2007). Pressure-induced and store-operated cation influx in vascular smooth muscle cells is independent of TRPC1. Pflug. Arch..

[71] Gottlieb P., Folgering J., Maroto R., Raso A., Wood T.G., Kurosky A., Bowman C., Bichet D., Patel A., Sachs F. (2008). Revisiting TRPC1 and TRPC6 mechanosensitivity. Pflug. Arch..

[72] Staaf S., Maxvall I., Lind U., Husmark J., Mattsson J.P., Ernfors P., Pierrou S. (2009). Down regulation of TRPC1 by shRNA reduces mechanosensitivity in mouse dorsal root ganglion neurons in vitro. Neurosci. Lett..

[73] Garrison S.R., Dietrich A., Stucky C.L. (2012). TRPC1 contributes to light-touch sensation and mechanical responses in low-threshold cutaneous sensory neurons. J. Neurophysiol..

[74] Alessandri-Haber N., Dina O.A., Chen X., Levine J.D. (2009). TRPC1 and TRPC6 channels cooperate with TRPV4 to mediate mechanical hyperalgesia and nociceptor sensitization. J. Neurosci..

[75] Semtner M., Schaefer M., Pinkenburg O., Plant T.D. (2007). Potentiation of TRPC5 by protons. J. Biol. Chem..

[76] Obukhov A.G., Nowycky M.C. (2008). TRPC5 channels undergo changes in gating properties during the activation-deactivation cycle. J. Cell Physiol..

[77] Strubing C., Krapivinsky G., Krapivinsky L., Clapham D.E. (2001). TRPC1 and TRPC5 form a novel cation channel in mammalian brain. Neuron.

[78] Hofmann T., Schaefer M., Schultz G., Gudermann T. (2002). Subunit composition of mammalian transient receptor potential channels in living cells. Proc. Natl. Acad. Sci. USA.

[79] Obukhov A.G., Nowycky M.C. (2005). A cytosolic residue mediates Mg^2+^ block and regulates inward current amplitude of a transient receptor potential channel. J. Neurosci..

[80] Strubing C., Krapivinsky G., Krapivinsky L., Clapham D.E. (2003). Formation of novel TRPC channels by complex subunit interactions in embryonic brain. J. Biol. Chem..

[81] Yip H., Chan W.Y., Leung P.C., Kwan H.Y., Liu C., Huang Y., Michel V., Yew D.T., Yao X. (2004). Expression of TRPC homologs in endothelial cells and smooth muscle layers of human arteries. Histochem. Cell Biol..

[82] Freichel M., Vennekens R., Olausson J., Stolz S., Philipp S.E., Weissgerber P., Flockerzi V. (2005). Functional role of TRPC proteins in native systems: Implications from knockout and knock-down studies. J. Physiol..

[83] Ku C.Y., Babich L., Word R.A., Zhong M., Ulloa A., Monga M., Sanborn B.M. (2006). Expression of transient receptor channel proteins in human fundal myometrium in pregnancy. J. Soc. Gynecol. Investig..

[84] Kunert-Keil C., Bisping F., Kruger J., Brinkmeier H. (2006). Tissue-specific expression of TRP channel genes in the mouse and its variation in three different mouse strains. BMC Genom..

[85] Elg S., Marmigere F., Mattsson J.P., Ernfors P. (2007). Cellular subtype distribution and developmental regulation of TRPC channel members in the mouse dorsal root ganglion. J. Comp. Neurol..

[86] Wuensch T., Thilo F., Krueger K., Scholze A., Ristow M., Tepel M. (2010). High glucose-induced oxidative stress increases transient receptor potential channel expression in human monocytes. Diabetes.

[87] Xu P., Xu J., Li Z., Yang Z. (2012). Expression of TRPC6 in renal cortex and hippocampus of mouse during postnatal development. PLoS ONE.

[88] Xu S.Z., Beech D.J. (2001). TrpC1 is a membrane-spanning subunit of store-operated Ca(2+) channels in native vascular smooth muscle cells. Circ. Res..

[89] Startek J.B., Boonen B., Talavera K., Meseguer V. (2019). TRP Channels as Sensors of Chemically-Induced Changes in Cell Membrane Mechanical Properties. Int. J. Mol. Sci..

[90] Beech D.J., Muraki K., Flemming R. (2004). Non-selective cationic channels of smooth muscle and the mammalian homologues of Drosophila TRP. J. Physiol..

[91] Hu G., Oboukhova E.A., Kumar S., Sturek M., Obukhov A.G. (2009). Canonical transient receptor potential channels expression is elevated in a porcine model of metabolic syndrome. Mol. Endocrinol..

[92] Kumar S., Chakraborty S., Barbosa C., Brustovetsky T., Brustovetsky N., Obukhov A.G. (2012). Mechanisms controlling neurite outgrowth in a pheochromocytoma cell line: The role of TRPC channels. J. Cell Physiol..

[93] Tai Y., Yang S., Liu Y., Shao W. (2017). TRPC Channels in Health and Disease. Adv. Exp. Med. Biol..

[94] Winn M.P., Conlon P.J., Lynn K.L., Farrington M.K., Creazzo T., Hawkins A.F., Daskalakis N., Kwan S.Y., Ebersviller S., Burchette J.L. (2005). A mutation in the TRPC6 cation channel causes familial focal segmental glomerulosclerosis. Science.

[95] Reiser J., Polu K.R., Moller C.C., Kenlan P., Altintas M.M., Wei C., Faul C., Herbert S., Villegas I., Avila-Casado C. (2005). TRPC6 is a glomerular slit diaphragm-associated channel required for normal renal function. Nat. Genet..

[96] Yu Y., Keller S.H., Remillard C.V., Safrina O., Nicholson A., Zhang S.L., Jiang W., Vangala N., Landsberg J.W., Wang J.Y. (2009). A functional single-nucleotide polymorphism in the TRPC6 gene promoter associated with idiopathic pulmonary arterial hypertension. Circulation.

[97] Fan C., Choi W., Sun W., Du J., Lu W. (2018). Structure of the human lipid-gated cation channel TRPC3. Elife.

[98] Sierra-Valdez F., Azumaya C.M., Romero L.O., Nakagawa T., Cordero-Morales J.F. (2018). Structure-function analyses of the ion channel TRPC3 reveal that its cytoplasmic domain allosterically modulates channel gating. J. Biol. Chem..

[99] Tang Q., Guo W., Zheng L., Wu J.X., Liu M., Zhou X., Zhang X., Chen L. (2018). Structure of the receptor-activated human TRPC6 and TRPC3 ion channels. Cell Res..

[100] Azumaya C.M., Sierra-Valdez F., Cordero-Morales J.F., Nakagawa T. (2018). Cryo-EM structure of the cytoplasmic domain of murine transient receptor potential cation channel subfamily C member 6 (TRPC6). J. Biol. Chem..

[101] Vinayagam D., Mager T., Apelbaum A., Bothe A., Merino F., Hofnagel O., Gatsogiannis C., Raunser S. (2018). Electron cryo-microscopy structure of the canonical TRPC4 ion channel. Elife.

[102] Duan J., Li J., Zeng B., Chen G.L., Peng X., Zhang Y., Wang J., Clapham D.E., Li Z., Zhang J. (2018). Structure of the mouse TRPC4 ion channel. Nat. Commun..

[103] Duan J., Li J., Chen G.L., Ge Y., Liu J., Xie K., Peng X., Zhou W., Zhong J., Zhang Y. (2019). Cryo-EM structure of TRPC5 at 2.8-A resolution reveals unique and conserved structural elements essential for channel function. Sci. Adv..

[104] Poteser M., Schleifer H., Lichtenegger M., Schernthaner M., Stockner T., Kappe C.O., Glasnov T.N., Romanin C., Groschner K. (2011). PKC-dependent coupling of calcium permeation through transient receptor potential canonical 3 (TRPC3) to calcineurin signaling in HL-1 myocytes. Proc. Natl. Acad. Sci. USA.

[105] Lichtenegger M., Stockner T., Poteser M., Schleifer H., Platzer D., Romanin C., Groschner K. (2013). A novel homology model of TRPC3 reveals allosteric coupling between gate and selectivity filter. Cell Calcium.

[106] Xu S.Z., Sukumar P., Zeng F., Li J., Jairaman A., English A., Naylor J., Ciurtin C., Majeed Y., Milligan C.J. (2008). TRPC channel activation by extracellular thioredoxin. Nature.

[107] Hong C., Kwak M., Myeong J., Ha K., Wie J., Jeon J.H., So I. (2015). Extracellular disulfide bridges stabilize TRPC5 dimerization, trafficking, and activity. Pflug. Arch..

[108] Xu S.Z., Zeng F., Lei M., Li J., Gao B., Xiong C., Sivaprasadarao A., Beech D.J. (2005). Generation of functional ion-channel tools by E3 targeting. Nat. Biotechnol..

[109] Vinayagam D.Q.D., Sitsel O., Merino F., Stabrin M., Hofnagel O., Yu M., Ledeboer M.W., Malojcic G., Raunser S. (2020). Structural basis of TRPC4 regulation by calmodulin and pharmacological agents. bioRxiv.

[110] Zhu M.X. (2005). Multiple roles of calmodulin and other Ca(2+)-binding proteins in the functional regulation of TRP channels. Pflug. Arch..

[111] Liu C.H., Gong Z., Liang Z.L., Liu Z.X., Yang F., Sun Y.J., Ma M.L., Wang Y.J., Ji C.R., Wang Y.H. (2017). Arrestin-biased AT1R agonism induces acute catecholamine secretion through TRPC3 coupling. Nat. Commun..

[112] Song T., Hao Q., Zheng Y.M., Liu Q.H., Wang Y.X. (2015). Inositol 1,4,5-trisphosphate activates TRPC3 channels to cause extracellular Ca2+ influx in airway smooth muscle cells. Am. J. Physiol. Lung Cell Mol. Physiol..

[113] Kim H., Jeon J.P., Hong C., Kim J., Myeong J., Jeon J.H., So I. (2013). An essential role of PI(4,5)P(2) for maintaining the activity of the transient receptor potential canonical (TRPC)4beta. Pflug. Arch..

[114] Imai Y., Itsuki K., Okamura Y., Inoue R., Mori M.X. (2012). A self-limiting regulation of vasoconstrictor-activated TRPC3/C6/C7 channels coupled to PI(4,5)P(2)-diacylglycerol signalling. J. Physiol..

[115] Saleh S.N., Albert A.P., Large W.A. (2009). Activation of native TRPC1/C5/C6 channels by endothelin-1 is mediated by both PIP3 and PIP2 in rabbit coronary artery myocytes. J. Physiol..

[116] Itsuki K., Imai Y., Hase H., Okamura Y., Inoue R., Mori M.X. (2014). PLC-mediated PI(4,5)P2 hydrolysis regulates activation and inactivation of TRPC6/7 channels. J. Gen. Physiol..

[117] Myeong J., Kwak M., Jeon J.P., Hong C., Jeon J.H., So I. (2015). Close spatio-association of the transient receptor potential canonical 4 (TRPC4) channel with Galphai in TRPC4 activation process. Am. J. Physiol. Cell Physiol..

[118] Thakur D.P., Tian J.B., Jeon J., Xiong J., Huang Y., Flockerzi V., Zhu M.X. (2016). Critical roles of Gi/o proteins and phospholipase C-delta1 in the activation of receptor-operated TRPC4 channels. Proc. Natl. Acad. Sci. USA.

[119] Zimmermann J., Latta L., Beck A., Leidinger P., Fecher-Trost C., Schlenstedt G., Meese E., Wissenbach U., Flockerzi V. (2014). Trans-activation response (TAR) RNA-binding protein 2 is a novel modulator of transient receptor potential canonical 4 (TRPC4) protein. J. Biol. Chem..

[120] Bousquet S.M., Monet M., Boulay G. (2010). Protein kinase C-dependent phosphorylation of transient receptor potential canonical 6 (TRPC6) on serine 448 causes channel inhibition. J. Biol. Chem..

[121] Chen X., Egly C., Riley A.M., Li W., Tewson P., Hughes T.E., Quinn A.M., Obukhov A.G. (2014). PKC-dependent Phosphorylation of the H1 Histamine Receptor Modulates TRPC6 Activity. Cells.

[122] Hagmann H., Mangold N., Rinschen M.M., Koenig T., Kunzelmann K., Schermer B., Benzing T., Brinkkoetter P.T. (2018). Proline-dependent and basophilic kinases phosphorylate human TRPC6 at serine 14 to control channel activity through increased membrane expression. FASEB J..

[123] Zhang X., Trebak M. (2014). Transient receptor potential canonical 7: A diacylglycerol-activated non-selective cation channel. Handb. Exp. Pharmacol..

[124] DeHaven W.I., Jones B.F., Petranka J.G., Smyth J.T., Tomita T., Bird G.S., Putney J.W. (2009). TRPC channels function independently of STIM1 and Orai1. J. Physiol..

[125] Berridge M.J., Bootman M.D., Lipp P. (1998). Calcium—A life and death signal. Nature.

[126] Broker-Lai J., Kollewe A., Schindeldecker B., Pohle J., Nguyen Chi V., Mathar I., Guzman R., Schwarz Y., Lai A., Weissgerber P. (2017). Heteromeric channels formed by TRPC1, TRPC4 and TRPC5 define hippocampal synaptic transmission and working memory. EMBO J..

[127] Neuner S.M., Wilmott L.A., Hope K.A., Hoffmann B., Chong J.A., Abramowitz J., Birnbaumer L., O’Connell K.M., Tryba A.K., Greene A.S. (2015). TRPC3 channels critically regulate hippocampal excitability and contextual fear memory. Behav. Brain Res..

[128] Du J., Ma X., Shen B., Huang Y., Birnbaumer L., Yao X. (2014). TRPV4, TRPC1, and TRPP2 assemble to form a flow-sensitive heteromeric channel. FASEB J..

[129] Chen M.S., Xiao J.H., Wang Y., Xu B.M., Gao L., Wang J.L. (2013). Upregulation of TRPC1 contributes to contractile function in isoproterenol-induced hypertrophic myocardium of rat. Cell Physiol. Biochem..

[130] Seo K., Rainer P.P., Shalkey Hahn V., Lee D.I., Jo S.H., Andersen A., Liu T., Xu X., Willette R.N., Lepore J.J. (2014). Combined TRPC3 and TRPC6 blockade by selective small-molecule or genetic deletion inhibits pathological cardiac hypertrophy. Proc. Natl. Acad. Sci. USA.

[131] Han J.W., Lee Y.H., Yoen S.I., Abramowitz J., Birnbaumer L., Lee M.G., Kim J.Y. (2016). Resistance to pathologic cardiac hypertrophy and reduced expression of CaV1.2 in Trpc3-depleted mice. Mol. Cell Biochem..

[132] Sunggip C., Shimoda K., Oda S., Tanaka T., Nishiyama K., Mangmool S., Nishimura A., Numaga-Tomita T., Nishida M. (2018). TRPC5-eNOS Axis Negatively Regulates ATP-Induced Cardiomyocyte Hypertrophy. Front. Pharmacol..

[133] Cheng H., Li J., Wu Q., Zheng X., Gao Y., Yang Q., Sun N., He M., Zhou Y. (2020). Effect of SKF96365 on cardiomyocyte hypertrophy induced by angiotensin II. Mol. Med. Rep..

[134] Tang L., Yao F., Wang H., Wang X., Shen J., Dai B., Wu H., Zhou D., Guo F., Wang J. (2019). Inhibition of TRPC1 prevents cardiac hypertrophy via NF-kappaB signaling pathway in human pluripotent stem cell-derived cardiomyocytes. J. Mol. Cell Cardiol.

[135] Sabourin J., Boet A., Rucker-Martin C., Lambert M., Gomez A.M., Benitah J.P., Perros F., Humbert M., Antigny F. (2018). Ca(2+) handling remodeling and STIM1L/Orai1/TRPC1/TRPC4 upregulation in monocrotaline-induced right ventricular hypertrophy. J. Mol. Cell Cardiol.

[136] Doleschal B., Primessnig U., Wolkart G., Wolf S., Schernthaner M., Lichtenegger M., Glasnov T.N., Kappe C.O., Mayer B., Antoons G. (2015). TRPC3 contributes to regulation of cardiac contractility and arrhythmogenesis by dynamic interaction with NCX1. Cardiovasc. Res..

[137] Ju Y.K., Lee B.H., Trajanovska S., Hao G., Allen D.G., Lei M., Cannell M.B. (2015). The involvement of TRPC3 channels in sinoatrial arrhythmias. Front. Physiol..

[138] Yamaguchi Y., Iribe G., Kaneko T., Takahashi K., Numaga-Tomita T., Nishida M., Birnbaumer L., Naruse K. (2018). TRPC3 participates in angiotensin II type 1 receptor-dependent stress-induced slow increase in intracellular Ca(2+) concentration in mouse cardiomyocytes. J. Physiol. Sci..

[139] Eder P., Probst D., Rosker C., Poteser M., Wolinski H., Kohlwein S.D., Romanin C., Groschner K. (2007). Phospholipase C-dependent control of cardiac calcium homeostasis involves a TRPC3-NCX1 signaling complex. Cardiovasc. Res..

[140] Camacho Londono J.E., Tian Q., Hammer K., Schroder L., Camacho Londono J., Reil J.C., He T., Oberhofer M., Mannebach S., Mathar I. (2015). A background Ca^2+^ entry pathway mediated by TRPC1/TRPC4 is critical for development of pathological cardiac remodelling. Eur. Heart J..

[141] Washburn D.G., Holt D.A., Dodson J., McAtee J.J., Terrell L.R., Barton L., Manns S., Waszkiewicz A., Pritchard C., Gillie D.J. (2013). The discovery of potent blockers of the canonical transient receptor channels, TRPC3 and TRPC6, based on an anilino-thiazole pharmacophore. Bioorg. Med. Chem. Lett..

[142] Welsh D.G., Morielli A.D., Nelson M.T., Brayden J.E. (2002). Transient receptor potential channels regulate myogenic tone of resistance arteries. Circ. Res..

[143] Gonzales A.L., Yang Y., Sullivan M.N., Sanders L., Dabertrand F., Hill-Eubanks D.C., Nelson M.T., Earley S. (2014). A PLCgamma1-dependent, force-sensitive signaling network in the myogenic constriction of cerebral arteries. Sci. Signal..

[144] Alvarez-Miguel I., Cidad P., Perez-Garcia M.T., Lopez-Lopez J.R. (2017). Differences in TRPC3 and TRPC6 channels assembly in mesenteric vascular smooth muscle cells in essential hypertension. J. Physiol..

[145] Dietrich A., Mederos Y.S.M., Gollasch M., Gross V., Storch U., Dubrovska G., Obst M., Yildirim E., Salanova B., Kalwa H. (2005). Increased vascular smooth muscle contractility in TRPC6*−/−* mice. Mol. Cell Biol..

[146] Shi J., Miralles F., Birnbaumer L., Large W.A., Albert A.P. (2016). Store depletion induces Galphaq-mediated PLCbeta1 activity to stimulate TRPC1 channels in vascular smooth muscle cells. FASEB J..

[147] Shi J., Miralles F., Birnbaumer L., Large W.A., Albert A.P. (2017). Store-operated interactions between plasmalemmal STIM1 and TRPC1 proteins stimulate PLCbeta1 to induce TRPC1 channel activation in vascular smooth muscle cells. J. Physiol..

[148] Avila-Medina J., Calderon-Sanchez E., Gonzalez-Rodriguez P., Monje-Quiroga F., Rosado J.A., Castellano A., Ordonez A., Smani T. (2016). Orai1 and TRPC1 Proteins Co-localize with CaV1.2 Channels to Form a Signal Complex in Vascular Smooth Muscle Cells. J. Biol. Chem..

[149] Lemos V.S., Poburko D., Liao C.H., Cole W.C., van Breemen C. (2007). Na^+^ entry via TRPC6 causes Ca2+ entry via NCX reversal in ATP stimulated smooth muscle cells. Biochem. Biophys. Res. Commun..

[150] Zulian A., Baryshnikov S.G., Linde C.I., Hamlyn J.M., Ferrari P., Golovina V.A. (2010). Upregulation of Na^+^/Ca^2+^ exchanger and TRPC6 contributes to abnormal Ca2+ homeostasis in arterial smooth muscle cells from Milan hypertensive rats. Am. J. Physiol. Heart Circ. Physiol.

[151] Rosker C., Graziani A., Lukas M., Eder P., Zhu M.X., Romanin C., Groschner K. (2004). Ca(2+) signaling by TRPC3 involves Na(+) entry and local coupling to the Na(+)/Ca(2+) exchanger. J. Biol. Chem..

[152] Kraft R. (2007). The Na^+^/Ca^2+^ exchange inhibitor KB-R7943 potently blocks TRPC channels. Biochem. Biophys. Res. Commun..

[153] Li W., Chen X., Riley A.M., Hiett S.C., Temm C.J., Beli E., Long X., Chakraborty S., Alloosh M., White F.A. (2017). Long-term spironolactone treatment reduces coronary TRPC expression, vasoconstriction, and atherosclerosis in metabolic syndrome pigs. Basic Res. Cardiol..

[154] Numaga-Tomita T., Shimauchi T., Oda S., Tanaka T., Nishiyama K., Nishimura A., Birnbaumer L., Mori Y., Nishida M. (2019). TRPC6 regulates phenotypic switching of vascular smooth muscle cells through plasma membrane potential-dependent coupling with PTEN. FASEB J..

[155] Koenig S., Schernthaner M., Maechler H., Kappe C.O., Glasnov T.N., Hoefler G., Braune M., Wittchow E., Groschner K. (2013). A TRPC3 blocker, ethyl-1-(4-(2,3,3-trichloroacrylamide)phenyl)-5-(trifluoromethyl)-1H-pyrazole-4-c arboxylate (Pyr3), prevents stent-induced arterial remodeling. J. Pharmacol. Exp. Ther..

[156] Kumar B., Dreja K., Shah S.S., Cheong A., Xu S.Z., Sukumar P., Naylor J., Forte A., Cipollaro M., McHugh D. (2006). Upregulated TRPC1 channel in vascular injury in vivo and its role in human neointimal hyperplasia. Circ. Res..

[157] Liu D., Yang D., He H., Chen X., Cao T., Feng X., Ma L., Luo Z., Wang L., Yan Z. (2009). Increased transient receptor potential canonical type 3 channels in vasculature from hypertensive rats. Hypertension.

[158] Ma T., Lin S., Wang B., Wang Q., Xia W., Zhang H., Cui Y., He C., Wu H., Sun F. (2019). TRPC3 deficiency attenuates high salt-induced cardiac hypertrophy by alleviating cardiac mitochondrial dysfunction. Biochem. Biophys. Res. Commun..

[159] Wang B., Xiong S., Lin S., Xia W., Li Q., Zhao Z., Wei X., Lu Z., Wei X., Gao P. (2017). Enhanced Mitochondrial Transient Receptor Potential Channel, Canonical Type 3-Mediated Calcium Handling in the Vasculature From Hypertensive Rats. J. Am. Heart Assoc..

[160] Schleifer H., Doleschal B., Lichtenegger M., Oppenrieder R., Derler I., Frischauf I., Glasnov T.N., Kappe C.O., Romanin C., Groschner K. (2012). Novel pyrazole compounds for pharmacological discrimination between receptor-operated and store-operated Ca(2+) entry pathways. Br. J. Pharmacol..

[161] Weissmann N., Dietrich A., Fuchs B., Kalwa H., Ay M., Dumitrascu R., Olschewski A., Storch U., Mederos y Schnitzler M., Ghofrani H.A. (2006). Classical transient receptor potential channel 6 (TRPC6) is essential for hypoxic pulmonary vasoconstriction and alveolar gas exchange. Proc. Natl. Acad. Sci. USA.

[162] Malczyk M., Erb A., Veith C., Ghofrani H.A., Schermuly R.T., Gudermann T., Dietrich A., Weissmann N., Sydykov A. (2017). The Role of Transient Receptor Potential Channel 6 Channels in the Pulmonary Vasculature. Front. Immunol..

[163] Malczyk M., Veith C., Fuchs B., Hofmann K., Storch U., Schermuly R.T., Witzenrath M., Ahlbrecht K., Fecher-Trost C., Flockerzi V. (2013). Classical transient receptor potential channel 1 in hypoxia-induced pulmonary hypertension. Am. J. Respir. Crit. Care Med..

[164] Xia Y., Yang X.R., Fu Z., Paudel O., Abramowitz J., Birnbaumer L., Sham J.S. (2014). Classical transient receptor potential 1 and 6 contribute to hypoxic pulmonary hypertension through differential regulation of pulmonary vascular functions. Hypertension.

[165] Freichel M., Suh S.H., Pfeifer A., Schweig U., Trost C., Weissgerber P., Biel M., Philipp S., Freise D., Droogmans G. (2001). Lack of an endothelial store-operated Ca^2+^ current impairs agonist-dependent vasorelaxation in TRP4*−/−* mice. Nat. Cell Biol..

[166] Jung C., Gene G.G., Tomas M., Plata C., Selent J., Pastor M., Fandos C., Senti M., Lucas G., Elosua R. (2011). A gain-of-function SNP in TRPC4 cation channel protects against myocardial infarction. Cardiovasc. Res..

[167] Du L.L., Shen Z., Li Z., Ye X., Wu M., Hong L., Zhao Y. (2018). TRPC1 Deficiency Impairs the Endothelial Progenitor Cell Function via Inhibition of Calmodulin/eNOS Pathway. J. Cardiovasc. Transl. Res..

[168] Greenberg H.Z.E., Carlton-Carew S.R.E., Khan D.M., Zargaran A.K., Jahan K.S., Vanessa Ho W.S., Albert A.P. (2017). Heteromeric TRPV4/TRPC1 channels mediate calcium-sensing receptor-induced nitric oxide production and vasorelaxation in rabbit mesenteric arteries. Vascul. Pharmacol..

[169] Kuang C.Y., Yu Y., Wang K., Qian D.H., Den M.Y., Huang L. (2012). Knockdown of transient receptor potential canonical-1 reduces the proliferation and migration of endothelial progenitor cells. Stem Cells Dev..

[170] Yeon S.I., Kim J.Y., Yeon D.S., Abramowitz J., Birnbaumer L., Muallem S., Lee Y.H. (2014). Transient receptor potential canonical type 3 channels control the vascular contractility of mouse mesenteric arteries. PLoS ONE.

[171] Tauseef M., Knezevic N., Chava K.R., Smith M., Sukriti S., Gianaris N., Obukhov A.G., Vogel S.M., Schraufnagel D.E., Dietrich A. (2012). TLR4 activation of TRPC6-dependent calcium signaling mediates endotoxin-induced lung vascular permeability and inflammation. J. Exp. Med..

[172] Strielkov I., Krause N.C., Sommer N., Schermuly R.T., Ghofrani H.A., Grimminger F., Gudermann T., Dietrich A., Weissmann N. (2018). Hypoxic pulmonary vasoconstriction in isolated mouse pulmonary arterial vessels. Exp. Physiol..

[173] Silva J., Ballejo G. (2019). Pharmacological characterization of the calcium influx pathways involved in nitric oxide production by endothelial cells. Einstein (Sao Paulo).

[174] Miller M., Shi J., Zhu Y., Kustov M., Tian J.B., Stevens A., Wu M., Xu J., Long S., Yang P. (2011). Identification of ML204, a novel potent antagonist that selectively modulates native TRPC4/C5 ion channels. J. Biol. Chem..

[175] Villalba N., Sackheim A.M., Nunez I.A., Hill-Eubanks D.C., Nelson M.T., Wellman G.C., Freeman K. (2017). Traumatic Brain Injury Causes Endothelial Dysfunction in the Systemic Microcirculation through Arginase-1-Dependent Uncoupling of Endothelial Nitric Oxide Synthase. J. Neurotrauma.

[176] Chen X., Taylor-Nguyen N.N., Riley A.M., Herring B.P., White F.A., Obukhov A.G. (2019). The TRPC6 inhibitor, larixyl acetate, is effective in protecting against traumatic brain injury-induced systemic endothelial dysfunction. J. Neuroinflamm..

[177] Urban N., Wang L., Kwiek S., Rademann J., Kuebler W.M., Schaefer M. (2016). Identification and Validation of Larixyl Acetate as a Potent TRPC6 Inhibitor. Mol. Pharmacol..

[178] Tano J.Y., Solanki S., Lee R.H., Smedlund K., Birnbaumer L., Vazquez G. (2014). Bone marrow deficiency of TRPC3 channel reduces early lesion burden and necrotic core of advanced plaques in a mouse model of atherosclerosis. Cardiovasc. Res..

[179] Solanki S., Dube P.R., Birnbaumer L., Vazquez G. (2017). Reduced Necrosis and Content of Apoptotic M1 Macrophages in Advanced Atherosclerotic Plaques of Mice with Macrophage-Specific Loss of Trpc3. Sci. Rep..

[180] Smedlund K.B., Birnbaumer L., Vazquez G. (2015). Increased size and cellularity of advanced atherosclerotic lesions in mice with endothelial overexpression of the human TRPC3 channel. Proc. Natl. Acad. Sci. USA.

[181] Zhang Y., Qin W., Zhang L., Wu X., Du N., Hu Y., Li X., Shen N., Xiao D., Zhang H. (2015). MicroRNA-26a prevents endothelial cell apoptosis by directly targeting TRPC6 in the setting of atherosclerosis. Sci. Rep..

[182] Chaudhuri P., Rosenbaum M.A., Sinharoy P., Damron D.S., Birnbaumer L., Graham L.M. (2016). Membrane translocation of TRPC6 channels and endothelial migration are regulated by calmodulin and PI3 kinase activation. Proc. Natl. Acad. Sci. USA.

[183] Rosenbaum M.A., Chaudhuri P., Graham L.M. (2015). Hypercholesterolemia inhibits re-endothelialization of arterial injuries by TRPC channel activation. J. Vasc. Surg..

[184] Qi J., Cui J., Mi B., Yan X., Xu W., Ma H., Zhang Q., Xu F. (2020). Isoliquiritigenin Inhibits Atherosclerosis by Blocking TRPC5 Channel Expression. Cardiovasc. Ther..

[185] Liang C., Zhang Y., Zhuo D., Lo C.Y., Yu L., Lau C.W., Kwan Y.W., Tse G., Huang Y., Yao X. (2019). Endothelial cell transient receptor potential channel C5 (TRPC5) is essential for endothelium-dependent contraction in mouse carotid arteries. Biochem. Pharmacol..

[186] Gaunt H.J., Vasudev N.S., Beech D.J. (2016). Transient receptor potential canonical 4 and 5 proteins as targets in cancer therapeutics. Eur. Biophys. J..

[187] Asghar M.Y., Magnusson M., Kemppainen K., Sukumaran P., Lof C., Pulli I., Kalhori V., Tornquist K. (2015). Transient Receptor Potential Canonical 1 (TRPC1) Channels as Regulators of Sphingolipid and VEGF Receptor Expression: IMPLICATIONS FOR THYROID CANCER CELL MIGRATION AND PROLIFERATION. J. Biol. Chem..

[188] Ma X., Cai Y., He D., Zou C., Zhang P., Lo C.Y., Xu Z., Chan F.L., Yu S., Chen Y. (2012). Transient receptor potential channel TRPC5 is essential for P-glycoprotein induction in drug-resistant cancer cells. Proc. Natl. Acad. Sci. USA.

[189] Ma X., Chen Z., Hua D., He D., Wang L., Zhang P., Wang J., Cai Y., Gao C., Zhang X. (2014). Essential role for TrpC5-containing extracellular vesicles in breast cancer with chemotherapeutic resistance. Proc. Natl. Acad. Sci. USA.

[190] Zou Y., Chen M., Zhang S., Miao Z., Wang J., Lu X., Zhao X. (2019). TRPC5 induced autophagy promotes the TMZ resistance of glioma cells via the CAMMKbeta/AMPKalpha/mTOR pathway. Oncol. Rep..

[191] Sobradillo D., Hernandez-Morales M., Ubierna D., Moyer M.P., Nunez L., Villalobos C. (2014). A reciprocal shift in transient receptor potential channel 1 (TRPC1) and stromal interaction molecule 2 (STIM2) contributes to Ca2+ remodeling and cancer hallmarks in colorectal carcinoma cells. J. Biol. Chem..

[192] Gueguinou M., Harnois T., Crottes D., Uguen A., Deliot N., Gambade A., Chantome A., Haelters J.P., Jaffres P.A., Jourdan M.L. (2016). SK3/TRPC1/Orai1 complex regulates SOCE-dependent colon cancer cell migration: A novel opportunity to modulate anti-EGFR mAb action by the alkyl-lipid Ohmline. Oncotarget.

[193] Emmons M.F., Anreddy N., Cuevas J., Steinberger K., Yang S., McLaughlin M., Silva A., Hazlehurst L.A. (2017). MTI-101 treatment inducing activation of Stim1 and TRPC1 expression is a determinant of response in multiple myeloma. Sci. Rep..

[194] Tao X., Zhao N., Jin H., Zhang Z., Liu Y., Wu J., Bast R.C., Yu Y., Feng Y. (2013). FSH enhances the proliferation of ovarian cancer cells by activating transient receptor potential channel C3. Endocr. Relat. Cancer.

[195] Bernichtein S., Pigat N., Barry Delongchamps N., Boutillon F., Verkarre V., Camparo P., Reyes-Gomez E., Mejean A., Oudard S.M., Lepicard E.M. (2017). Vitamin D3 Prevents Calcium-Induced Progression of Early-Stage Prostate Tumors by Counteracting TRPC6 and Calcium Sensing Receptor Upregulation. Cancer Res..

[196] Jardin I., Diez-Bello R., Lopez J.J., Redondo P.C., Salido G.M., Smani T., Rosado J.A. (2018). TRPC6 Channels Are Required for Proliferation, Migration and Invasion of Breast Cancer Cell Lines by Modulation of Orai1 and Orai3 Surface Exposure. Cancers.

[197] Ding M., Wang H., Qu C., Xu F., Zhu Y., Lv G., Lu Y., Zhou Q., Zhou H., Zeng X. (2018). Pyrazolo[1,5-a]pyrimidine TRPC6 antagonists for the treatment of gastric cancer. Cancer Lett..

[198] Nielsen N., Kondratska K., Ruck T., Hild B., Kovalenko I., Schimmelpfennig S., Welzig J., Sargin S., Lindemann O., Christian S. (2017). TRPC6 channels modulate the response of pancreatic stellate cells to hypoxia. Pflug. Arch..

[199] Xu J., Zhang W., Cui W., Shi B., Wang H. (2019). PKCalpha promotes insulin secretion via TRPC1 phosphorylation in INS-1E cells. Biosci. Biotechnol. Biochem..

[200] Krout D., Schaar A., Sun Y., Sukumaran P., Roemmich J.N., Singh B.B., Claycombe-Larson K.J. (2017). The TRPC1 Ca(2+)-permeable channel inhibits exercise-induced protection against high-fat diet-induced obesity and type II diabetes. J. Biol. Chem..

[201] Chen K., Jin X., Li Q., Wang W., Wang Y., Zhang J. (2013). Association of TRPC1 gene polymorphisms with type 2 diabetes and diabetic nephropathy in Han Chinese population. Endocr. Res..

[202] Wang L., Chang J.H., Buckley A.F., Spurney R.F. (2019). Knockout of TRPC6 promotes insulin resistance and exacerbates glomerular injury in Akita mice. Kidney Int..

[203] Lanner J.T., Bruton J.D., Assefaw-Redda Y., Andronache Z., Zhang S.J., Severa D., Zhang Z.B., Melzer W., Zhang S.L., Katz A. (2009). Knockdown of TRPC3 with siRNA coupled to carbon nanotubes results in decreased insulin-mediated glucose uptake in adult skeletal muscle cells. FASEB J..

[204] Yamada H., Yoshida M., Ito K., Dezaki K., Yada T., Ishikawa S.E., Kakei M. (2016). Potentiation of Glucose-stimulated Insulin Secretion by the GPR40-PLC-TRPC Pathway in Pancreatic beta-Cells. Sci. Rep..

[205] Spires D., Ilatovskaya D.V., Levchenko V., North P.E., Geurts A.M., Palygin O., Staruschenko A. (2018). Protective role of Trpc6 knockout in the progression of diabetic kidney disease. Am. J. Physiol. Renal. Physiol..

[206] Ilatovskaya D.V., Blass G., Palygin O., Levchenko V., Pavlov T.S., Grzybowski M.N., Winsor K., Shuyskiy L.S., Geurts A.M., Cowley A.W. (2018). A NOX4/TRPC6 Pathway in Podocyte Calcium Regulation and Renal Damage in Diabetic Kidney Disease. J. Am. Soc. Nephrol..

[207] Sachdeva R., Schlotterer A., Schumacher D., Matka C., Mathar I., Dietrich N., Medert R., Kriebs U., Lin J., Nawroth P. (2018). TRPC proteins contribute to development of diabetic retinopathy and regulate glyoxalase 1 activity and methylglyoxal accumulation. Mol. Metab..

[208] Marabita F., Islam M.S. (2017). Expression of Transient Receptor Potential Channels in the Purified Human Pancreatic beta-Cells. Pancreas.

[209] Islam M.S. (2020). Molecular Regulations and Functions of the Transient Receptor Potential Channels of the Islets of Langerhans and Insulinoma Cells. Cells.

[210] Li M., Liu E., Zhou Q., Li S., Wang X., Liu Y., Wang L., Sun D., Ye J., Gao Y. (2018). TRPC1 Null Exacerbates Memory Deficit and Apoptosis Induced by Amyloid-beta. J. Alzheimers Dis..

[211] Martinez-Galan J.R., Verdejo A., Caminos E. (2018). TRPC1 Channels Are Expressed in Pyramidal Neurons and in a Subset of Somatostatin Interneurons in the Rat Neocortex. Front. Neuroanat..

[212] Wang D., Yu H., Xu B., Xu H., Zhang Z., Ren X., Yuan J., Liu J., Guo Y., Spencer P.S. (2018). TRPC1 Deletion Causes Striatal Neuronal Cell Apoptosis and Proteomic Alterations in Mice. Front. Aging Neurosci..

[213] Zhou J., Jia Y. (2017). TRPC Channels and Programmed Cell Death. Adv. Exp. Med. Biol..

[214] Chen C., Ma Q., Deng P., Yang J., Yang L., Lin M., Yu Z., Zhou Z. (2017). Critical role of TRPC1 in thyroid hormone-dependent dopaminergic neuron development. Biochim. Biophys. Acta Mol. Cell Res..

[215] Hao H.B., Webb S.E., Yue J., Moreau M., Leclerc C., Miller A.L. (2018). TRPC3 is required for the survival, pluripotency and neural differentiation of mouse embryonic stem cells (mESCs). Sci. China Life Sci..

[216] Stroh O., Freichel M., Kretz O., Birnbaumer L., Hartmann J., Egger V. (2012). NMDA receptor-dependent synaptic activation of TRPC channels in olfactory bulb granule cells. J. Neurosci..

[217] Greka A., Navarro B., Oancea E., Duggan A., Clapham D.E. (2003). TRPC5 is a regulator of hippocampal neurite length and growth cone morphology. Nat. Neurosci..

[218] Riccio A., Li Y., Moon J., Kim K.S., Smith K.S., Rudolph U., Gapon S., Yao G.L., Tsvetkov E., Rodig S.J. (2009). Essential role for TRPC5 in amygdala function and fear-related behavior. Cell.

[219] Phelan K.D., Shwe U.T., Abramowitz J., Wu H., Rhee S.W., Howell M.D., Gottschall P.E., Freichel M., Flockerzi V., Birnbaumer L. (2013). Canonical transient receptor channel 5 (TRPC5) and TRPC1/4 contribute to seizure and excitotoxicity by distinct cellular mechanisms. Mol. Pharmacol..

[220] Just S., Chenard B.L., Ceci A., Strassmaier T., Chong J.A., Blair N.T., Gallaschun R.J., Del Camino D., Cantin S., D’Amours M. (2018). Treatment with HC-070, a potent inhibitor of TRPC4 and TRPC5, leads to anxiolytic and antidepressant effects in mice. PLoS ONE.

[221] Ko A.R., Kang T.C. (2017). TRPC6-mediated ERK1/2 phosphorylation prevents dentate granule cell degeneration via inhibiting mitochondrial elongation. Neuropharmacology.

[222] Wu J., Ryskamp D., Birnbaumer L., Bezprozvanny I. (2018). Inhibition of TRPC1-Dependent Store-Operated Calcium Entry Improves Synaptic Stability and Motor Performance in a Mouse Model of Huntington’s Disease. J. Huntingtons Dis..

[223] Lepannetier S., Gualdani R., Tempesta S., Schakman O., Seghers F., Kreis A., Yerna X., Slimi A., de Clippele M., Tajeddine N. (2018). Activation of TRPC1 Channel by Metabotropic Glutamate Receptor mGluR5 Modulates Synaptic Plasticity and Spatial Working Memory. Front. Cell Neurosci..

[224] Yerna X., Schakman O., Ratbi I., Kreis A., Lepannetier S., de Clippele M., Achouri Y., Tajeddine N., Tissir F., Gualdani R. (2020). Role of the TRPC1 Channel in Hippocampal Long-Term Depression and in Spatial Memory Extinction. Int. J. Mol. Sci..

[225] Schwarz Y., Oleinikov K., Schindeldecker B., Wyatt A., Weissgerber P., Flockerzi V., Boehm U., Freichel M., Bruns D. (2019). TRPC channels regulate Ca2+-signaling and short-term plasticity of fast glutamatergic synapses. PLoS Biol..

[226] Klipec W.D., Burrow K.R., O’Neill C., Cao J.L., Lawyer C.R., Ostertag E., Fowler M., Bachtell R.K., Illig K.R., Cooper D.C. (2016). Loss of the trpc4 gene is associated with a reduction in cocaine self-administration and reduced spontaneous ventral tegmental area dopamine neuronal activity, without deficits in learning for natural rewards. Behav. Brain Res..

[227] Egorov A.V., Schumacher D., Medert R., Birnbaumer L., Freichel M., Draguhn A. (2019). TRPC channels are not required for graded persistent activity in entorhinal cortex neurons. Hippocampus.

[228] Chen X., Lu M., He X., Ma L., Birnbaumer L., Liao Y. (2017). TRPC3/6/7 Knockdown Protects the Brain from Cerebral Ischemia Injury via Astrocyte Apoptosis Inhibition and Effects on NF-small ka, CyrillicB Translocation. Mol. Neurobiol..

[229] Guo J., Li J., Xia L., Wang Y., Zhu J., Du J., Lu Y., Liu G., Yao X., Shen B. (2020). Transient Receptor Potential Canonical 5-Scramblase Signaling Complex Mediates Neuronal Phosphatidylserine Externalization and Apoptosis. Cells.

[230] Park S.E., Song J.H., Hong C., Kim D.E., Sul J.W., Kim T.Y., Seo B.R., So I., Kim S.Y., Bae D.J. (2019). Contribution of Zinc-Dependent Delayed Calcium Influx via TRPC5 in Oxidative Neuronal Death and its Prevention by Novel TRPC Antagonist. Mol. Neurobiol..

[231] Xu N., Meng H., Liu T., Feng Y., Qi Y., Wang H. (2018). TRPC1 Deficiency Exacerbates Cerebral Ischemia/Reperfusion-Induced Neurological Injury by Potentiating Nox4-Derived Reactive Oxygen Species Generation. Cell Physiol. Biochem..

[232] Griesi-Oliveira K., Acab A., Gupta A.R., Sunaga D.Y., Chailangkarn T., Nicol X., Nunez Y., Walker M.F., Murdoch J.D., Sanders S.J. (2015). Modeling non-syndromic autism and the impact of TRPC6 disruption in human neurons. Mol. Psychiatr..

[233] Hartmann J., Konnerth A. (2015). TRPC3-dependent synaptic transmission in central mammalian neurons. J. Mol. Med..

[234] Tian J., Zhu M.X. (2018). GABAB Receptors Augment TRPC3-Mediated Slow Excitatory Postsynaptic Current to Regulate Cerebellar Purkinje Neuron Response to Type-1 Metabotropic Glutamate Receptor Activation. Cells.

[235] Koizumi H., John T.T., Chia J.X., Tariq M.F., Phillips R.S., Mosher B., Chen Y., Thompson R., Zhang R., Koshiya N. (2018). Transient Receptor Potential Channels TRPM4 and TRPC3 Critically Contribute to Respiratory Motor Pattern Formation but not Rhythmogenesis in Rodent Brainstem Circuits. eNeuro.

[236] Phelan K.D., Shwe U.T., Abramowitz J., Birnbaumer L., Zheng F. (2014). Critical role of canonical transient receptor potential channel 7 in initiation of seizures. Proc. Natl. Acad. Sci. USA.

[237] Phelan K.D., Shwe U.T., Cozart M.A., Wu H., Mock M.M., Abramowitz J., Birnbaumer L., Zheng F. (2017). TRPC3 channels play a critical role in the theta component of pilocarpine-induced status epilepticus in mice. Epilepsia.

[238] Phelan K.D., Mock M.M., Kretz O., Shwe U.T., Kozhemyakin M., Greenfield L.J., Dietrich A., Birnbaumer L., Freichel M., Flockerzi V. (2012). Heteromeric canonical transient receptor potential 1 and 4 channels play a critical role in epileptiform burst firing and seizure-induced neurodegeneration. Mol. Pharmacol..

[239] Lewis A.H., Cui A.F., McDonald M.F., Grandl J. (2017). Transduction of Repetitive Mechanical Stimuli by Piezo1 and Piezo2 Ion Channels. Cell Rep..

[240] Zhang M., Wang Y., Geng J., Zhou S., Xiao B. (2019). Mechanically Activated Piezo Channels Mediate Touch and Suppress Acute Mechanical Pain Response in Mice. Cell Rep..

[241] Zhang X., Beckel J.M., Daugherty S.L., Wang T., Woodcock S.R., Freeman B.A., de Groat W.C. (2014). Activation of TRPC channels contributes to OA-NO2-induced responses in guinea-pig dorsal root ganglion neurons. J. Physiol..

[242] Alkhani H., Ase A.R., Grant R., O’Donnell D., Groschner K., Seguela P. (2014). Contribution of TRPC3 to store-operated calcium entry and inflammatory transductions in primary nociceptors. Mol. Pain.

[243] Alawi K.M., Russell F.A., Aubdool A.A., Srivastava S., Riffo-Vasquez Y., Baldissera L., Thakore P., Saleque N., Fernandes E.S., Walsh D.A. (2017). Transient receptor potential canonical 5 (TRPC5) protects against pain and vascular inflammation in arthritis and joint inflammation. Ann. Rheum. Dis..

[244] Vandewauw I., De Clercq K., Mulier M., Held K., Pinto S., Van Ranst N., Segal A., Voet T., Vennekens R., Zimmermann K. (2018). A TRP channel trio mediates acute noxious heat sensing. Nature.

[245] Sexton J.E., Desmonds T., Quick K., Taylor R., Abramowitz J., Forge A., Kros C.J., Birnbaumer L., Wood J.N. (2016). The contribution of TRPC1, TRPC3, TRPC5 and TRPC6 to touch and hearing. Neurosci. Lett..

[246] Dalrymple A., Slater D.M., Beech D., Poston L., Tribe R.M. (2002). Molecular identification and localization of Trp homologues, putative calcium channels, in pregnant human uterus. Mol. Hum. Reprod..

[247] Persoons E., Hennes A., De Clercq K., Van Bree R., Vriens G., O D.F., Peterse D., Vanhie A., Meuleman C., Voets T. (2018). Functional Expression of TRP Ion Channels in Endometrial Stromal Cells of Endometriosis Patients. Int. J. Mol. Sci..

[248] Kawarabayashi Y., Hai L., Honda A., Horiuchi S., Tsujioka H., Ichikawa J., Inoue R. (2012). Critical role of TRPC1-mediated Ca(2)(+) entry in decidualization of human endometrial stromal cells. Mol. Endocrinol..

[249] De Clercq K., Held K., Van Bree R., Meuleman C., Peeraer K., Tomassetti C., Voets T., D’Hooghe T., Vriens J. (2015). Functional expression of transient receptor potential channels in human endometrial stromal cells during the luteal phase of the menstrual cycle. Hum. Reprod..

[250] Sharma A., Nakade U.P., Choudhury S., Garg S.K. (2017). Functional involvement of protein kinase C, Rho-kinase and TRPC3 decreases while PLC increases with advancement of pregnancy in mediating oxytocin-induced myometrial contractions in water buffaloes (*Bubalus bubalis*). Theriogenology.

[251] Jing C., Dongming Z., Hong C., Quan N., Sishi L., Caixia L. (2018). TRPC3 Overexpression Promotes the Progression of Inflammation-Induced Preterm Labor and Inhibits T Cell Activation. Cell Physiol. Biochem..

[252] Hasna J., Abi Nahed R., Sergent F., Alfaidy N., Bouron A. (2019). The Deletion of TRPC6 Channels Perturbs Iron and Zinc Homeostasis and Pregnancy Outcome in Mice. Cell Physiol. Biochem..

[253] Gonzalez-Cobos J.C., Trebak M. (2010). TRPC channels in smooth muscle cells. Front. Biosci..

[254] Dwyer L., Rhee P.L., Lowe V., Zheng H., Peri L., Ro S., Sanders K.M., Koh S.D. (2011). Basally activated nonselective cation currents regulate the resting membrane potential in human and monkey colonic smooth muscle. Am. J. Physiol. Gastrointest Liver Physiol..

[255] Tsvilovskyy V.V., Zholos A.V., Aberle T., Philipp S.E., Dietrich A., Zhu M.X., Birnbaumer L., Freichel M., Flockerzi V. (2009). Deletion of TRPC4 and TRPC6 in mice impairs smooth muscle contraction and intestinal motility in vivo. Gastroenterology.

[256] Dryn D.O., Melnyk M.I., Al Kury L.T., Prylutskyy Y.I., Ritter U., Zholos A.V. (2018). C60 fullerenes disrupt cellular signalling leading to TRPC4 and TRPC6 channels opening by the activation of muscarinic receptors and G-proteins in small intestinal smooth muscles. Cell Signal..

[257] Dryn D., Luo J., Melnyk M., Zholos A., Hu H. (2018). Inhalation anaesthetic isoflurane inhibits the muscarinic cation current and carbachol-induced gastrointestinal smooth muscle contractions. Eur. J. Pharmacol..

[258] Schlondorff J. (2017). TRPC6 and kidney disease: Sclerosing more than just glomeruli?. Kidney Int..

[259] Heeringa S.F., Moller C.C., Du J., Yue L., Hinkes B., Chernin G., Vlangos C.N., Hoyer P.F., Reiser J., Hildebrandt F. (2009). A novel TRPC6 mutation that causes childhood FSGS. PLoS ONE.

[260] Mukerji N., Damodaran T.V., Winn M.P. (2007). TRPC6 and FSGS: The latest TRP channelopathy. Biochim. Biophys. Acta.

[261] Wilson C., Dryer S.E. (2014). A mutation in TRPC6 channels abolishes their activation by hypoosmotic stretch but does not affect activation by diacylglycerol or G protein signaling cascades. Am. J. Physiol. Renal. Physiol..

[262] Gheissari A., Meamar R., Kheirollahi M., Rouigari M., Dehbashi M., Dehghani L., Abedini A. (2018). TRPC6 Mutational Analysis in Iranian Children With Focal Segmental Glomerulosclerosis. Iran. J. Kidney Dis..

[263] Riehle M., Buscher A.K., Gohlke B.O., Kassmann M., Kolatsi-Joannou M., Brasen J.H., Nagel M., Becker J.U., Winyard P., Hoyer P.F. (2016). TRPC6 G757D Loss-of-Function Mutation Associates with FSGS. J. Am. Soc. Nephrol..

[264] Hall G., Wang L., Spurney R.F. (2019). TRPC Channels in Proteinuric Kidney Diseases. Cells.

[265] Zhou Y., Castonguay P., Sidhom E.H., Clark A.R., Dvela-Levitt M., Kim S., Sieber J., Wieder N., Jung J.Y., Andreeva S. (2017). A small-molecule inhibitor of TRPC5 ion channels suppresses progressive kidney disease in animal models. Science.

[266] Farmer L.K., Rollason R., Whitcomb D.J., Ni L., Goodliff A., Lay A.C., Birnbaumer L., Heesom K.J., Xu S.Z., Saleem M.A. (2019). TRPC6 Binds to and Activates Calpain, Independent of Its Channel Activity, and Regulates Podocyte Cytoskeleton, Cell Adhesion, and Motility. J. Am. Soc. Nephrol..

[267] Verheijden K.A.T., Sonneveld R., Bakker-van Bebber M., Wetzels J.F.M., van der Vlag J., Nijenhuis T. (2018). The Calcium-Dependent Protease Calpain-1 Links TRPC6 Activity to Podocyte Injury. J. Am. Soc. Nephrol..

[268] Schlondorff J., Del Camino D., Carrasquillo R., Lacey V., Pollak M.R. (2009). TRPC6 mutations associated with focal segmental glomerulosclerosis cause constitutive activation of NFAT-dependent transcription. Am. J. Physiol. Cell Physiol..

[269] Wang Q., Wang D., Shibata S., Ji T., Zhang L., Zhang R., Yang H., Ma L., Jiao J. (2019). Group I metabotropic glutamate receptor activation induces TRPC6-dependent calcium influx and RhoA activation in cultured human kidney podocytes. Biochem. Biophys. Res. Commun..

[270] Sun X., Chu Y., Zhang C., Du X., He F., Chen S., Gao P., Liu J., Zhu Z., Meng X. (2012). Effect of TRPC6 knockdown on puromycin aminonucleoside-induced podocyte injury. J. Huazhong Univ. Sci. Technol. Med. Sci..

[271] Kong W., Haschler T.N., Nurnberg B., Kramer S., Gollasch M., Marko L. (2019). Renal Fibrosis, Immune Cell Infiltration and Changes of TRPC Channel Expression after Unilateral Ureteral Obstruction in Trpc6*−/−* Mice. Cell Physiol. Biochem..

[272] Hou X., Xiao H., Zhang Y., Zeng X., Huang M., Chen X., Birnbaumer L., Liao Y. (2018). Transient receptor potential channel 6 knockdown prevents apoptosis of renal tubular epithelial cells upon oxidative stress via autophagy activation. Cell Death Dis..

[273] Wu Q.Y., Sun M.R., Wu C.L., Li Y., Du J.J., Zeng J.Y., Bi H.L., Sun Y.H. (2015). Activation of calcium-sensing receptor increases TRPC3/6 expression in T lymphocyte in sepsis. Mol. Immunol..

[274] Riazanski V., Gabdoulkhakova A.G., Boynton L.S., Eguchi R.R., Deriy L.V., Hogarth D.K., Loaec N., Oumata N., Galons H., Brown M.E. (2015). TRPC6 channel translocation into phagosomal membrane augments phagosomal function. Proc. Natl. Acad. Sci. USA.

[275] Pereira D.M.S., Mendes S.J.F., Alawi K., Thakore P., Aubdool A., Sousa N.C.F., da Silva J.F.R., Castro J.A., IC P.P., Silva L.C.N. (2018). Transient Receptor Potential Canonical Channels 4 and 5 Mediate Escherichia coli-Derived Thioredoxin Effects in Lipopolysaccharide-Injected Mice. Oxid Med. Cell Longev..

[276] Braun A., Varga-Szabo D., Kleinschnitz C., Pleines I., Bender M., Austinat M., Bosl M., Stoll G., Nieswandt B. (2009). Orai1 (CRACM1) is the platelet SOC channel and essential for pathological thrombus formation. Blood.

[277] Pulcinelli F.M., Trifiro E., Massimi I., Di Renzo L. (2013). A functional interaction between TRPC/NCKX induced by DAG plays a role in determining calcium influx independently from PKC activation. Platelets.

[278] Mahaut-Smith M.P. (2013). A role for platelet TRPC channels in the Ca^2+^ response that induces procoagulant activity. Sci. Signal..

[279] Albarran L., Berna-Erro A., Dionisio N., Redondo P.C., Lopez E., Lopez J.J., Salido G.M., Brull Sabate J.M., Rosado J.A. (2014). TRPC6 participates in the regulation of cytosolic basal calcium concentration in murine resting platelets. Biochim. Biophys. Acta.

[280] Gao Y., Yao T., Deng Z., Sohn J.W., Sun J., Huang Y., Kong X., Yu K.J., Wang R.T., Chen H. (2017). TrpC5 Mediates Acute Leptin and Serotonin Effects via Pomc Neurons. Cell Rep..

[281] Rode B., Yuldasheva N.Y., Baxter P.D., Sedo A., Ainscough J.F., Shires M., Kearney M.T., Bailey M.A., Wheatcroft S.B., Beech D.J. (2019). TRPC5 ion channel permeation promotes weight gain in hypercholesterolaemic mice. Sci. Rep..

[282] Wolfrum C., Kiehlmann E., Pelczar P. (2018). TRPC1 regulates brown adipose tissue activity in a PPARgamma-dependent manner. Am. J. Physiol. Endocrinol. Metab..

[283] Alawi K.M., Tandio D., Xu J., Thakore P., Papacleovoulou G., Fernandes E.S., Legido-Quigley C., Williamson C., Brain S.D. (2017). Transient receptor potential canonical 5 channels plays an essential role in hepatic dyslipidemia associated with cholestasis. Sci. Rep..

[284] Sel S., Rost B.R., Yildirim A.O., Sel B., Kalwa H., Fehrenbach H., Renz H., Gudermann T., Dietrich A. (2008). Loss of classical transient receptor potential 6 channel reduces allergic airway response. Clin. Exp. Allergy.

[285] Zhang X., Zhao Z., Ma L., Guo Y., Li X., Zhao L., Tian C., Tang X., Cheng D., Chen Z. (2018). The effects of transient receptor potential channel (TRPC) on airway smooth muscle cell isolated from asthma model mice. J. Cell Biochem..

[286] Pu Q., Zhao Y., Sun Y., Huang T., Lin P., Zhou C., Qin S., Singh B.B., Wu M. (2019). TRPC1 intensifies house dust mite-induced airway remodeling by facilitating epithelial-to-mesenchymal transition and STAT3/NF-kappaB signaling. FASEB J..

[287] Kepura F., Braun E., Dietrich A., Plant T.D. (2020). TRPC1 Regulates the Activity of a Voltage-Dependent Nonselective Cation Current in Hippocampal CA1 Neurons. Cells.

[288] Oda S., Numaga-Tomita T., Kitajima N., Toyama T., Harada E., Shimauchi T., Nishimura A., Ishikawa T., Kumagai Y., Birnbaumer L. (2017). TRPC6 counteracts TRPC3-Nox2 protein complex leading to attenuation of hyperglycemia-induced heart failure in mice. Sci. Rep..

[289] Nunez L., Bird G.S., Hernando-Perez E., Perez-Riesgo E., Putney J.W., Villalobos C. (2019). Store-operated Ca(2+) entry and Ca(2+) responses to hypothalamic releasing hormones in anterior pituitary cells from Orai1*−/−* and heptaTRPC knockout mice. Biochim. Biophys. Acta Mol. Cell Res..

[290] Wang H., Cheng X., Tian J., Xiao Y., Tian T., Xu F., Hong X., Zhu M.X. (2020). TRPC channels: Structure, function, regulation and recent advances in small molecular probes. Pharmacol. Ther..

[291] Rubaiy H.N. (2019). Treasure troves of pharmacological tools to study transient receptor potential canonical 1/4/5 channels. Br. J. Pharmacol..

[292] Minard A., Bauer C.C., Wright D.J., Rubaiy H.N., Muraki K., Beech D.J., Bon R.S. (2018). Remarkable Progress with Small-Molecule Modulation of TRPC1/4/5 Channels: Implications for Understanding the Channels in Health and Disease. Cells.

[293] Bon R.S., Beech D.J. (2013). In pursuit of small molecule chemistry for calcium-permeable non-selective TRPC channels -- mirage or pot of gold?. Br. J. Pharmacol..

[294] Kiyonaka S., Kato K., Nishida M., Mio K., Numaga T., Sawaguchi Y., Yoshida T., Wakamori M., Mori E., Numata T. (2009). Selective and direct inhibition of TRPC3 channels underlies biological activities of a pyrazole compound. Proc. Natl. Acad. Sci. USA.

[295] Miller M.R., Shi J., Wu M., Engers J., Hopkins C.R., Lindsley C.W., Salovich J.M., Zhu Y., Tian J.B., Zhu M.X. (2010). Novel Chemical Inhibitor of TRPC4 Channels. Probe Reports from the NIH Molecular Libraries Program.

[296] Akbulut Y., Gaunt H.J., Muraki K., Ludlow M.J., Amer M.S., Bruns A., Vasudev N.S., Radtke L., Willot M., Hahn S. (2015). (-)-Englerin A is a potent and selective activator of TRPC4 and TRPC5 calcium channels. Angew. Chem. Int. Ed. Engl..

[297] Maruyama T., Kanaji T., Nakade S., Kanno T., Mikoshiba K. (1997). 2APB, 2-aminoethoxydiphenyl borate, a membrane-penetrable modulator of Ins(1,4,5)P3-induced Ca^2^+ release. J. Biochem..

[298] Prakriya M., Lewis R.S. (2001). Potentiation and inhibition of Ca(2+) release-activated Ca(2+) channels by 2-aminoethyldiphenyl borate (2-APB) occurs independently of IP(3) receptors. J. Physiol..

[299] Jairaman A., Prakriya M. (2013). Molecular pharmacology of store-operated CRAC channels. Channels (Austin).

[300] Hu H.Z., Gu Q., Wang C., Colton C.K., Tang J., Kinoshita-Kawada M., Lee L.Y., Wood J.D., Zhu M.X. (2004). 2-aminoethoxydiphenyl borate is a common activator of TRPV1, TRPV2, and TRPV3. J. Biol. Chem..

[301] Xu S.Z., Zeng F., Boulay G., Grimm C., Harteneck C., Beech D.J. (2005). Block of TRPC5 channels by 2-aminoethoxydiphenyl borate: A differential, extracellular and voltage-dependent effect. Br. J. Pharmacol..

[302] Merritt J.E., Armstrong W.P., Benham C.D., Hallam T.J., Jacob R., Jaxa-Chamiec A., Leigh B.K., McCarthy S.A., Moores K.E., Rink T.J. (1990). SK&F 96365, a novel inhibitor of receptor-mediated calcium entry. Biochem. J..

[303] Singh A., Hildebrand M.E., Garcia E., Snutch T.P. (2010). The transient receptor potential channel antagonist SKF96365 is a potent blocker of low-voltage-activated T-type calcium channels. Br. J. Pharmacol..

[304] Bai Y., Yu X., Chen H., Horne D., White R., Wu X., Lee P., Gu Y., Ghimire-Rijal S., Lin D.C. (2020). Structural basis for pharmacological modulation of the TRPC6 channel. Elife.

[305] de la Cruz G.G., Svobodova B., Lichtenegger M., Tiapko O., Groschner K., Glasnov T. (2017). Intensified Microwave-Assisted N-Acylation Procedure—Synthesis and Activity Evaluation of TRPC3 Channel Agonists with a 1,3-Dihydro-2H-benzo[d]imidazol-2-one Core. Synlett.

[306] Qu C., Ding M., Zhu Y., Lu Y., Du J., Miller M., Tian J., Zhu J., Xu J., Wen M. (2017). Pyrazolopyrimidines as Potent Stimulators for Transient Receptor Potential Canonical 3/6/7 Channels. J. Med. Chem..

[307] Tiapko O., Shrestha N., Lindinger S., Guedes de la Cruz G., Graziani A., Klec C., Butorac C., Graier W.F., Kubista H., Freichel M. (2019). Lipid-independent control of endothelial and neuronal TRPC3 channels by light. Chem. Sci..

[308] Leinders-Zufall T., Storch U., Bleymehl K., Mederos Y.S.M., Frank J.A., Konrad D.B., Trauner D., Gudermann T., Zufall F. (2018). PhoDAGs Enable Optical Control of Diacylglycerol-Sensitive Transient Receptor Potential Channels. Cell Chem. Biol..

[309] Urban N., Schaefer M. (2020). Direct Activation of TRPC3 Channels by the Antimalarial Agent Artemisinin. Cells.

[310] Motoyama K., Nagata T., Kobayashi J., Nakamura A., Miyoshi N., Kazui M., Sakurai K., Sakakura T. (2018). Discovery of a bicyclo[4.3.0]nonane derivative DS88790512 as a potent, selective, and orally bioavailable blocker of transient receptor potential canonical 6 (TRPC6). Bioorg. Med. Chem. Lett..

[311] Lin B.L., Matera D., Doerner J.F., Zheng N., Del Camino D., Mishra S., Bian H., Zeveleva S., Zhen X., Blair N.T. (2019). In vivo selective inhibition of TRPC6 by antagonist BI 749327 ameliorates fibrosis and dysfunction in cardiac and renal disease. Proc. Natl. Acad. Sci. USA.

[312] Rubaiy H.N., Ludlow M.J., Bon R.S., Beech D.J. (2017). Pico145—Powerful new tool for TRPC1/4/5 channels. Channels (Austin).

[313] Naylor J., Minard A., Gaunt H.J., Amer M.S., Wilson L.A., Migliore M., Cheung S.Y., Rubaiy H.N., Blythe N.M., Musialowski K.E. (2016). Natural and synthetic flavonoid modulation of TRPC5 channels. Br. J. Pharmacol..

[314] Maier T., Follmann M., Hessler G., Kleemann H.W., Hachtel S., Fuchs B., Weissmann N., Linz W., Schmidt T., Lohn M. (2015). Discovery and pharmacological characterization of a novel potent inhibitor of diacylglycerol-sensitive TRPC cation channels. Br. J. Pharmacol..

[315] Hafner S., Burg F., Kannler M., Urban N., Mayer P., Dietrich A., Trauner D., Broichhagen J., Schaefer M. (2018). A (+)-Larixol Congener with High Affinity and Subtype Selectivity toward TRPC6. ChemMedChem.

